# Evolution shapes ecosystem resilience through spatial self-organization

**DOI:** 10.1073/pnas.2536473123

**Published:** 2026-10-12

**Authors:** Xiaoli Dong, Alexander J. Richardson, Rachata Muneepeerakul

**Affiliations:** ^a^ Department of Environmental Science and Policy, University of California, Davis, CA 95616; ^b^ Department of Mathematics, University of California, Davis, CA 95616; ^c^ Department of Agricultural and Biological Engineering, University of Florida, Gainesville, FL 32611

**Keywords:** drylands, pattern formation, multistability, rainfall variability, stochasticity

## Abstract

Drylands are globally widespread and ecologically important but vulnerable to climate change. Many exhibit spatially self-organized vegetation patterns that enhance resilience by enabling vegetation to reorganize their patterns under drying, thereby optimizing water use and maintaining productivity. Plants can also undergo evolution in response to environmental change. Although both spatial self-organization and rapid evolution are fundamental mechanisms of resilience, their interaction remains largely unknown. Using a theoretical model, we examine how these mechanisms interact to shape vegetation transitions—abrupt or gradual—and ecosystem resilience under climatic drying. We find that pattern formation can suppress adaptation by maintaining soil moisture beneath vegetation patches despite rainfall decline, thereby limiting selection for increased drought tolerance. However, this suppression is largely alleviated under the variable rainfall typical of drylands. In turn, evolution feeds back on self-organization by increasing the spatial plasticity of vegetation patterns and their ability to reorganize under changing climate, although this positive effect weakens as evolutionary rate increases. Considering direct (adaptation) and indirect (pattern-mediated) effects, evolution enhances resilience, with indirect effects via pattern plasticity dominating. Within model scenarios spanning plausible rainfall variability and relative evolutionary-to-ecological rates, more than half of vegetation transitions are abrupt involving biomass losses exceeding 50%, highlighting a potential mechanism underlying recent global observations of widespread abrupt vegetation losses in drylands. These results demonstrate that evolution and spatial self-organization jointly determine dryland resilience under environmental change and highlight the unexpected role of evolution in patterned landscapes, calling for integrative approaches to predict ecosystem responses in a changing world.

## Significance

When ecosystems face climate change, they respond in multiple ways. In drylands, vegetation adjusts its spatial patterns responding to increasing water stress, while plants may rapidly evolve greater drought tolerance. How these responses interact to affect dryland resilience remains unclear. We show that vegetation pattern formation limits evolutionary adaptation, although this constraint is reduced under the variable rainfall typical of drylands. In turn, evolution increases how flexibly vegetation patterns adjust to environmental change, but this benefit weakens when evolution is faster. Overall, evolution increases resilience mainly by enhancing the flexibility of vegetation patterns, while its direct effect through increased drought tolerance plays a smaller role. These findings highlight the importance of integrating evolution and pattern formation when predicting dryland changes.

Drylands are globally widespread and are ecologically significant but are especially sensitive to climate change (1, 2). Vegetation in drylands is spatially patchy due to severe water limitation. Many vegetation spatial patterns in drylands are self-organized, a process wherein regular patterns emerge from local interactions (3–5). These regular patterns, such as bands, gaps, and mazes, have been commonly observed in dryland ecosystems around the globe (4, 6, 7). By redistributing the limiting resources (e.g., water), spatial self-organization acts to increase the resilience of ecosystems. As the environment becomes increasingly drier, vegetation undergoes a sequence of pattern transitions as a response (8). Such spatial plasticity allows ecosystems to more efficiently use the limiting resource (water) and thereby delay the collapse of ecosystems into the bare soil state. As such, spatial self-organization serves as an important mechanism by which ecosystems buffer against environmental change (8–10).

Rapid evolution is another mechanism by which ecosystems respond to climate change (11, 12). Rapid evolution occurs at a similar timescale to that of ecological dynamics, often in the range of a few decades (12). Increasing evidence indicates that organisms can adapt to environmental change by rapid evolution—by increasing the frequency of genes with higher fitness in the changed environment, thus enabling the population to persist under increasingly stressful conditions (13–16). For example, traits that support dehydration avoidance, such as smaller, thicker leaves, evolved in scarlet monkey flower (*Mimulus cardinalis*) after a recent 7-y drought in California (17). As two fundamental mechanisms, spatial self-organization and rapid evolution very likely operate together as climate dries. However, little research has been done so far on their relative role in shaping dryland resilience under global change or on how they interact to alter their mutual effectiveness.

Spatial self-organization and evolution may interact in complex ways (18, 19). A recent theoretical study by Bera at al. suggests that vegetation pattern formation inhibits evolutionary adaptations (20). This effect is attributed to the high spatial plasticity of vegetation patterns—through self-organized patch thinning (reducing vegetation patch size) and coarsening (reducing patch number), ecosystems can maintain local water availability despite declining rainfall. This stress-induced rapid adjustment of spatial patterns can reduce the exposure of populations to environmental change and inhibit evolutionary adaptation to the stress. However, their model assumes that evolutionary responses are much slower than ecological processes (20). It remains unclear whether, when evolution operates at the similar time scales to those of vegetation pattern formation, adaptation will no longer be inhibited and could play a more substantial role in maintaining dryland ecosystems under increasing aridity.

Evolutionary homeostasis in spatially self-organized ecosystems is based on predictions of pattern formation models that the spatial plasticity of patterns can maintain soil moisture beneath vegetation patches, thereby shielding plants from natural selection (20). Yet, numerous dryland studies report strong fluctuations of soil moisture in vegetation patches under the intense rainfall interannual variability typical in arid/semiarid regions (21, 22). Moreover, soil moisture at the edges of vegetation patches is less buffered by plant-soil moisture feedbacks [plant induced changes in soil water in turn affects plant growth (21, 23)] and closely tracks ambient environment (24). Although adaptation is often perceived to be a large-scale process, recent findings reveal adaptive divergence at fine patch scales regardless of high gene flow (25, 26). Together, temporal and spatial variability may sustain opportunities for evolution in drylands with patterned vegetation.

Evolution could, in turn, alter vegetation pattern transitions and resilience (27). Adaptations to drought may enable populations to persist under drier conditions, reducing the need for patch thinning or coarsening and potentially delaying—or even suppressing—pattern transitions. However, maintaining dense vegetation under increasing aridity could raise the risk of sudden, early ecosystem collapse, thereby reducing resilience (28). Evolution may also introduce trait heterogeneity among individuals, reducing the regularity of patch spacing. Spatial regularity strongly governs pattern transitions (28, 29)—regular patterns tend to lose half the patches abruptly, whereas irregular patterns typically lose patches one at a time, leading to frequent incremental pattern adjustments as rainfall declines and higher system adaptability and resilience. How evolution affects the plasticity of patterns remains unresolved but critical for both pattern transitions and resilience—First, recent theory predicts gradual vegetation transitions via coexisting patterned states (10), whereas empirical evidence shows widespread abrupt shifts globally (30): can evolution reconcile this discrepancy through its potential effect on pattern plasticity? Second, does evolution ultimately enhance or reduce resilience, given that reduced plasticity may decrease pattern adaptability (28), while evolutionary adaptation promotes persistence of individual plants?

Here, we investigate how spatial self-organization and evolution interact to shape dryland responses to climatic drying, with emphasis on how evolution affects the resilience of patterned vegetation. We extend a recent vegetation pattern formation model with evolutionary dynamics (20) by incorporating i) stochastic rainfall and trait variation among individuals to capture fine-scale local adaptation, and ii) variable rates of evolution to represent ecoevolutionary dynamics. We find that evolutionary adaptation can occur in patterned landscapes and enhances dryland resilience, measured as the rainfall threshold below which biomass collapses. However, this effect depends on evolutionary rate: although evolution enhances resilience, its contribution can weaken at higher rates, where vegetation transitions are increasingly dominated by large, abrupt shifts. Notably, evolution influences resilience primarily by altering the spatial plasticity of vegetation patterns, less through direct adaptive trait changes.

## Results

### Mathematical Modeling of Dryland Vegetation.

Vegetation patterning in drylands arises from scale-dependent feedbacks: enhanced infiltration beneath vegetation induces overland water flow toward vegetated patches, reinforcing growth while depleting surrounding areas (23, 31, 32). This combination of local positive and long-range negative feedbacks generate spatial patterns that persist under lower rainfall than uniform vegetation, as patches draw additional water from adjacent bare soil. We describe these dynamics using a well-established, one-dimensional reaction–diffusion model (9, 20, 33):

[1] $$\begin{array}{c} \partial_{t}B=\underset{\text{growth}}{\underbrace{c\Gamma \left(1-\frac{B}{K}\right)WB}}\;\;\;-\underset{\text{mortality}}{\underbrace{MB}}+\underset{\text{dispersal}}{\underbrace{D_{B}\partial_{x}^{2}B}}, \end{array}$$

[2] $$\partial_{t}W=\underset{\text{infiltration}}{\underbrace{A\frac{YB+fQ}{YB+Q}H}}-\underset{\text{evaporation}}{\underbrace{L_{0}W}}-\underset{\text{plant}\;\text{uptake}}{\underbrace{\Gamma WB}}+\underset{\text{diffusion}}{\underbrace{D_{W}\partial_{x}^{2}W}},$$

[3] $$\partial_{t}H=\underset{\text{rainfall}}{\underbrace{P}}-\underset{\text{infiltration}}{\underbrace{A\frac{YB+fQ}{YB+Q}H}}+\underset{\text{diffusion}}{\underbrace{D_{H}\partial_{x}^{2}H}},$$

where *B* is the biomass density (kg m^−2^) and *W* and *H* are soil water and overland water level (mm), respectively. *B* changes by plant growth, mortality, and dispersal, captured by a diffusion term (Eq. **1**). *W* is governed by infiltration, evaporation, plant uptake, and soil water diffusion (Eq. **2**). Overland water level, *H*, depends on rainfall input, infiltration, and surface water transport, modeled as a diffusion process for simplicity (34) (Eq. **3**).

In addition to driving vegetation pattern formation, water stress can induce evolutionary adaptation. Following Bera et al. (20), we represent this adaptation with a quantitative trait χ∈[0,1] that governs a trade-off between plant investment in growth and drought tolerance:

[4a] $$K=K_{\max}+\chi \left(K_{\min}-K_{\max}\right),$$

[4b] $$M=M_{\max}+\chi \left(M_{\min}-M_{\max}\right),$$

where *K* is the carrying capacity and *M* is the mortality rate (Eq. **1**). Increasing χ shifts plant investment toward drought tolerance, reducing mortality but also lowering carrying capacity, thereby capturing a commonly observed growth–mortality trade-off (35–37). A variety of plant traits could underlie this tradeoff, such as plant increasing the fraction of root mass while reducing fractions of stem, leaf, and reproductive mass or reducing leaf nitrogen content under drying (38). We model a single species, so χ represents within-population trait variation, and evolutionary dynamics reflect shifts in the frequency of trait-associated genes in a population without invoking speciation.

In contrast to Bera et al. (20), who assume a spatially uniform trait value χ (*Materials and Methods*), we allow χ to vary in space. Trait dynamics follow a continuous-space quantitative genetic model (39), describing both dispersal-driven gene flow and directional selection arising local selection gradient (25) (derivation in *SI Appendix*, section S1):

[5a] $$\partial_{t}\chi =\underset{\text{genetic drift}\;\text{driven}\;\text{by}\;\text{dispersal}}{\underbrace{2D_{B}\frac{\partial \text{ln}B}{\partial x}\frac{\partial \chi }{\partial x}+D_{B}\partial_{x}^{2}\chi }}+\underset{\text{directional}\;\text{selection}}{\underbrace{C_{\chi }\frac{\partial r}{\partial \chi }}},$$

[5b] $$r=\frac{1}{B}\frac{\partial B}{\partial t},$$

where *r* is the per capita growth rate and ∂r/∂χ is the local selection gradient. The additive genetic variance $C_{\chi }$ determines the rate of evolutionary response, with larger $C_{\chi }$ leading to faster adaptation (39). We consider a broad range of $C_{\chi }$ (~1 to 50% of ecological rates; *Materials and Methods*) to assess the role of evolutionary timescales. Parameter definitions and values are given in *SI Appendix*, Table S1; additional model details and numerical methods are provided in the *Materials and Methods*.

We explored model behavior across a rainfall gradient (15 to 135 mm y^−1^; in 5 mm increments) using 2,500 simulations (25 rainfall levels × 100 replicates). This range covers a broad spectrum of rainfall conditions relevant to drylands, while replication captures multistability—multiple stable spatial patterns under the same rainfall (10, 40). Each simulation was first run to equilibrium from random initial conditions. Then we imposed progressive drying under scenarios with and without rainfall interannual variability, and with evolution active or paused.

### Pattern Formation Shapes Evolutionarily Stable States.

To assess the effect of pattern formation, we compared equilibrium states with and without vegetation pattern formation. When the infiltration contrast parameter f≪1 (Eqs. **2** and **3**), infiltration is enhanced beneath vegetation, drawing surface water from adjacent bare soil and generating the scale-dependent feedbacks that drive pattern formation. In contrast, setting f=1 removes infiltration contrast: lateral water redistribution ceases and pattern formation is suppressed.

**Fig. 1.**
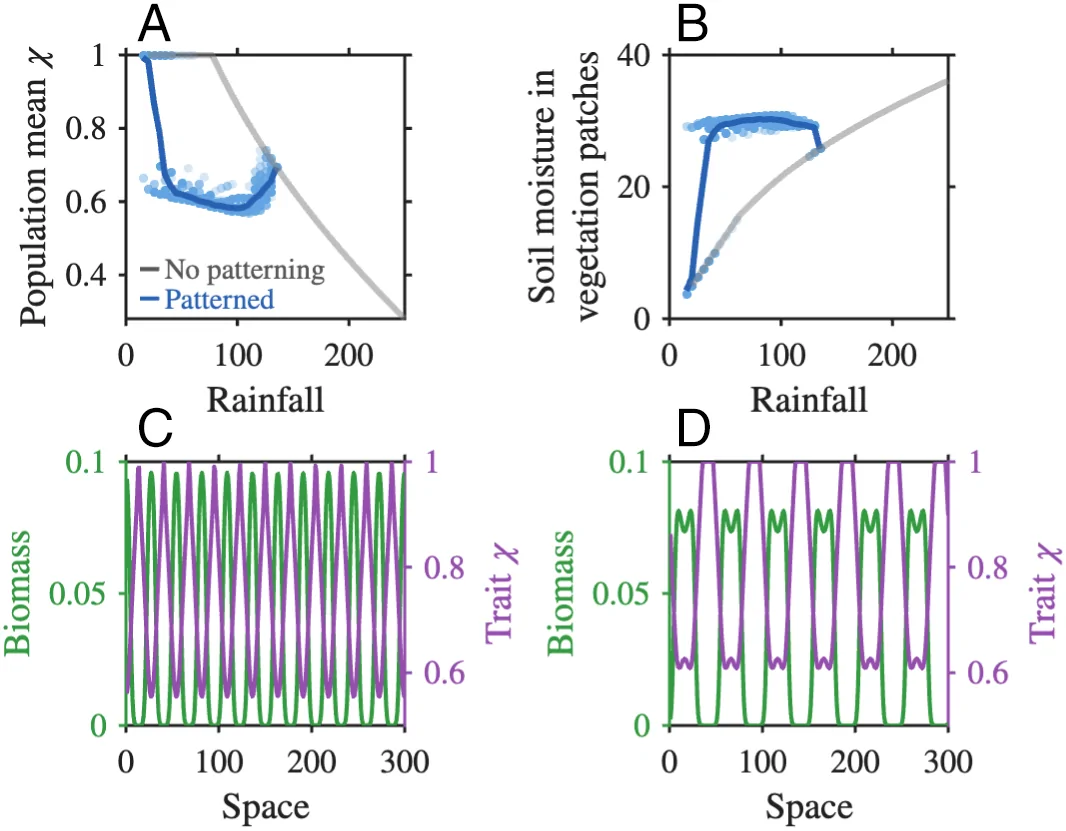
Steady state solutions for vegetation pattern formation and ecosystem multistability. (*A* and *B*) Steady-state population mean χ and soil moisture within vegetation patches with pattern formation (blue) and without (gray). For pattern-forming cases, 100 simulations were run at each rainfall level (15 to 135 mm y^−1^, in 5 mm increments) to capture multistability. Points denote individual simulations and lines indicate mean across replicates. (*C* and *D*) Example of multistability: at *P* = 85 mm y^−1^, ecosystems stabilize at distinct vegetation spatial patterns and evolutionary states. Parameter: $C_{\chi }$ = 0.050.

In patterned landscapes, populations stabilize within a narrow range of χ across a wide rainfall gradient (Fig. 1*A*). This occurs because local feedbacks maintain consistently high soil moisture within vegetation patches (Fig. 1*B*). In contrast, without patterning (f=1), mean χ increases linearly with aridity (Fig. 1*A*) and stabilizes at much higher values (greater drought tolerance). Consequently, populations at sites with markedly different rainfall can converge to similar trait values. Conversely, for a given rainfall level, populations may stabilize at different trait values because ecosystem multistability permits multiple stable vegetation patterns (10, 40) and corresponding evolutionarily stable states (Fig. 1 *C* and *D* and *SI Appendix*, Fig. S1). Together, these results indicate that pattern formation can decouple evolutionary stable states from environmental conditions.

### Self-Organization Inhibits Evolutionary Responses to Drying.

**Fig. 2.**
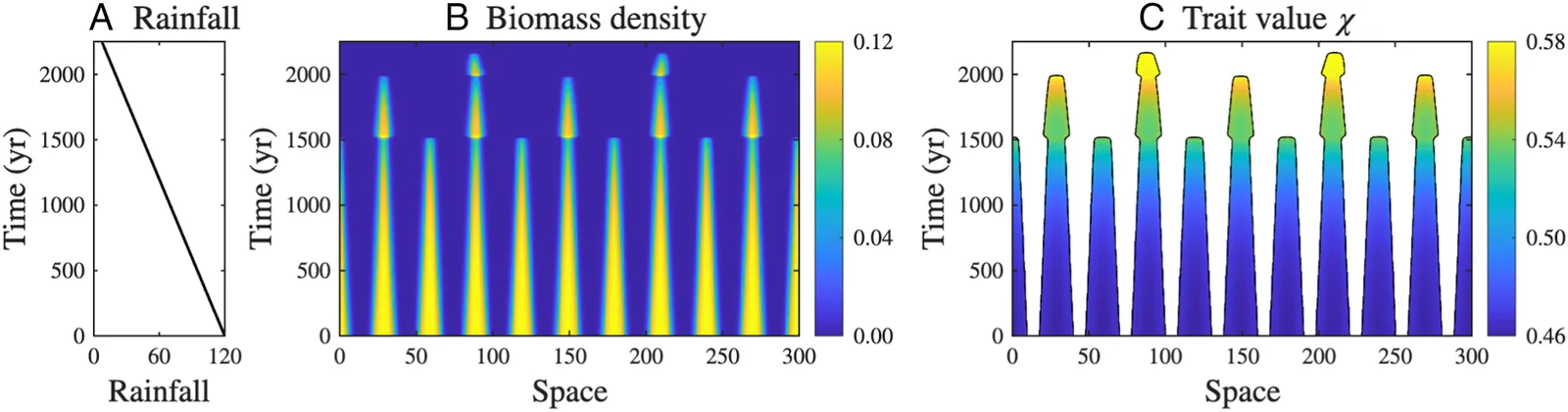
Transition of vegetation spatial patterns and evolution of trait χ under drying. (*A*) Temporal trajectory of rainfall *P*. (*B*) Changes of vegetation spatial pattern, showing patch thinning and coarsening following the onset of drying (year 0). (*C*) Corresponding spatial and temporal dynamics of trait χ. White regions indicate bare soils (biomass density < 0.002), and the black contours delineate vegetation patch boundaries. Parameters: $C_{\chi }=0.01$, *C_P_* = 0.05 mm y^−2^, initial *P* = 120 mm y^−1^.

**Fig. 3.**
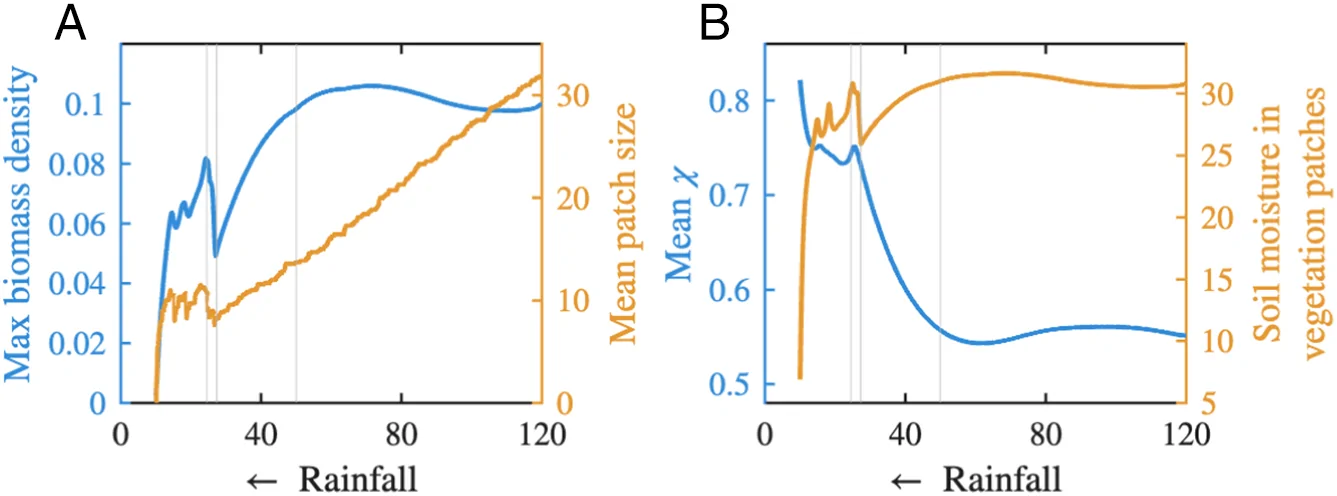
Three phases in the response of patterned vegetation to climatic drying. (*A*) Maximum biomass density, (*B*) soil moisture within vegetation patches, and population mean χ remain approximately constant as rainfall declines to ~55 mm y^−1^, while patch size declines linearly (Phase I, buffered thinning). Below this threshold, maximum biomass density and soil moisture decline with rainfall, driving rapid adaptive evolution in χ (Phase II, reactive thinning). In Phase III, coarsening occurs, resetting the evolutionary state. Parameters: $C_{\chi }=0.010$, *C_P_* = 0.1 mm y^−2^, initial *P* = 120 mm y^−1^.

We next examine dynamics under a drying climate, with rates of rainfall decline typical of drylands (41–43) (*Materials and Methods*). Patterned vegetation responds through three recurring phases (Figs. 2 and 3; Movie S1). In Phase I (buffered thinning), reductions in patch size compensate for declining rainfall, maintaining nearly constant conditions (e.g., soil moisture, biomass density) within vegetation patches (Fig. 3), resulting in little evolutionary change (Figs. 2*C* and 3*B*). In Phase II (reactive thinning), vegetation patches continue to thin, but soil moisture and biomass density in them begin to decline (Figs. 2*B* and 3), triggering rapid evolution toward greater drought tolerance (Figs. 2*C* and 3*B*). In Phase III (coarsening), some patches disappear, and the system reorganizes into patterns with larger interpatch spacing (Fig. 2*B*). The resulting reduction in vegetation cover increases soil moisture in remaining patches, driving evolution toward lower drought tolerance despite continued drying (Fig. 3*B*).

This sequence repeats as drying continues (Figs. 2 and 3). Through spatial plasticity—pattern thinning and coarsening—vegetation persists at much lower rainfall than without pattern formation (compare *SI Appendix*, Fig. S2 *A* and *D*). However, the same plasticity constrains evolutionary adaptation. Thinning continuously buffers soil moisture within vegetation patches, limiting selection for increased drought tolerance (Figs. 2*C* and 3*B*). Meanwhile, coarsening acts as a punctuated reset, reducing vegetation cover while elevating soil moisture in remaining patches, disrupting or even temporarily reversing adaptive trends (Fig. 3*B*). Without pattern formation, the population mean χ increases linearly with declining rainfall (*SI Appendix*, Fig. S2*F*). Notably, this inhibition does not arise because evolution is slower than spatial self-organization; it persists even when both processes operate on similar timescales (*SI Appendix*, Fig. S2*C*).

### Rainfall Stochasticity Weakens Inhibition on Evolution.

**Fig. 4.**
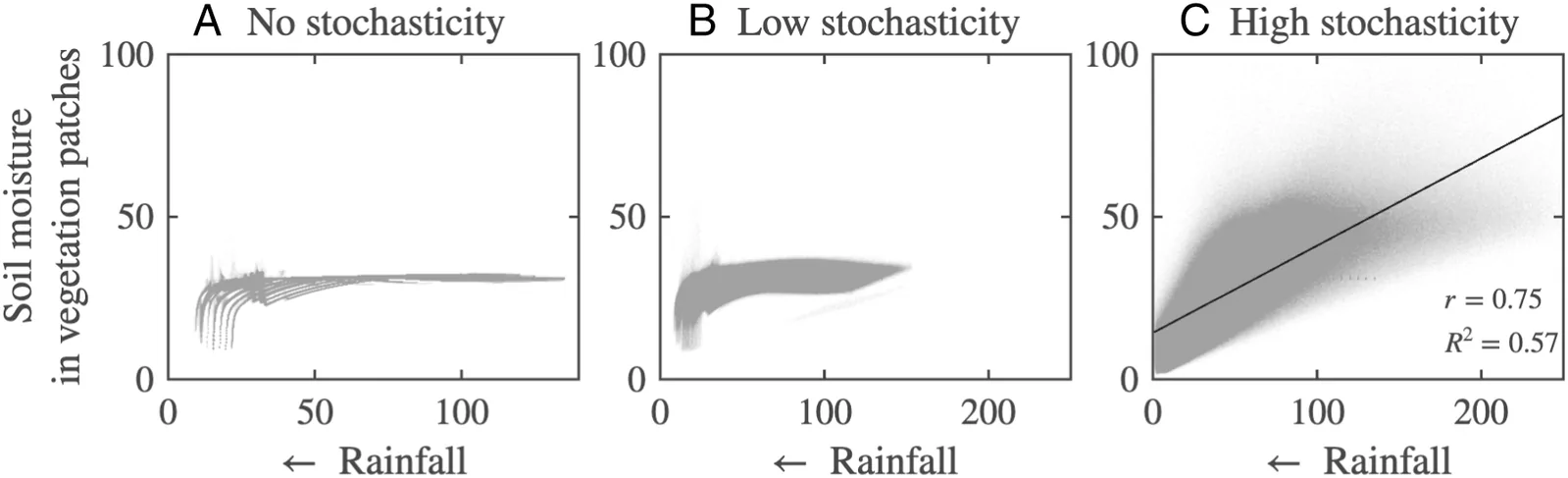
Rainfall stochasticity enhances correlations between soil moisture in vegetation patches and rainfall. (*A*) In the absence of stochasticity, soil moisture within vegetation patches remains stable across a wide range of rainfall conditions. (*B* and *C*) As stochasticity increases (coefficient of variation of annual rainfall = 0.08 and 0.54, respectively), the correlation between undercanopy moisture and rainfall increases. *r,* Pearson correlation coefficient; *R^2^*, proportion of variance in soil moisture explained by rainfall. Each panel summarizes 2,500 simulations. Parameters: $C_{\chi }=0.010$, *C_P_* = 0.05 mm y^−2^.

Next, we incorporated rainfall interannual variability typical of drylands into the model (*Materials and Methods*) to examine how environmental stochasticity modifies the inhibitory effects of spatial self-organization on evolution. Stochasticity weakens the stabilizing effects of spatial plasticity of vegetation patterns, allowing soil moisture within patches to “feel” ambient rainfall (Fig. 4). Across 2,500 simulations, undercanopy soil moisture remains strongly correlated with rainfall during climatic drying (*r* = 0.75; Fig. 4*C*), consistent with field observations of pronounced year-to-year variability in dryland soil moisture (21, 22). Without stochasticity, soil moisture in vegetation patches remains unrealistically stable (Fig. 4*A*). Increasing variability strengthens this rainfall-moisture coupling (Fig. 4). Consistent with these ensemble-level results, the same relationship holds within individual simulations: higher interannual variability leads to closer tracking of undercanopy soil moisture with rainfall (*SI Appendix*, Fig. S3).

### Evolution Alters Pattern Plasticity and Vegetation Transitions.

**Fig. 5.**
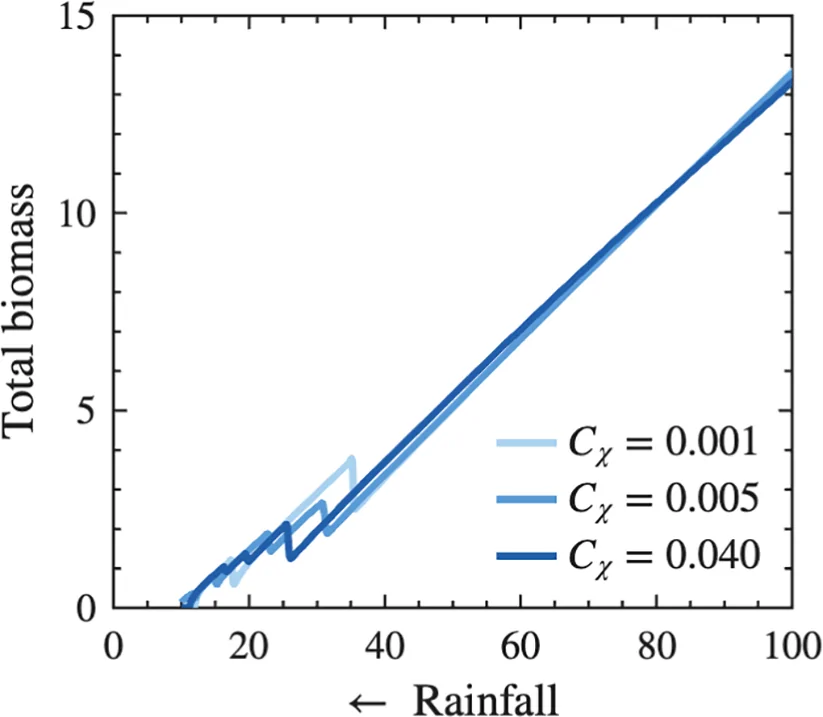
Evolution delays vegetation coarsening during climatic drying. Coarsening, indicated by abrupt increases in total biomass, occurs at lower rainfall levels when evolution is faster (higher $C_{\chi }$). Results are shown without rainfall stochasticity. Parameters: $C_{P}$ = 0.025 mm y^−2^, initial *P* = 100 mm y^−1^.

**Fig. 6.**
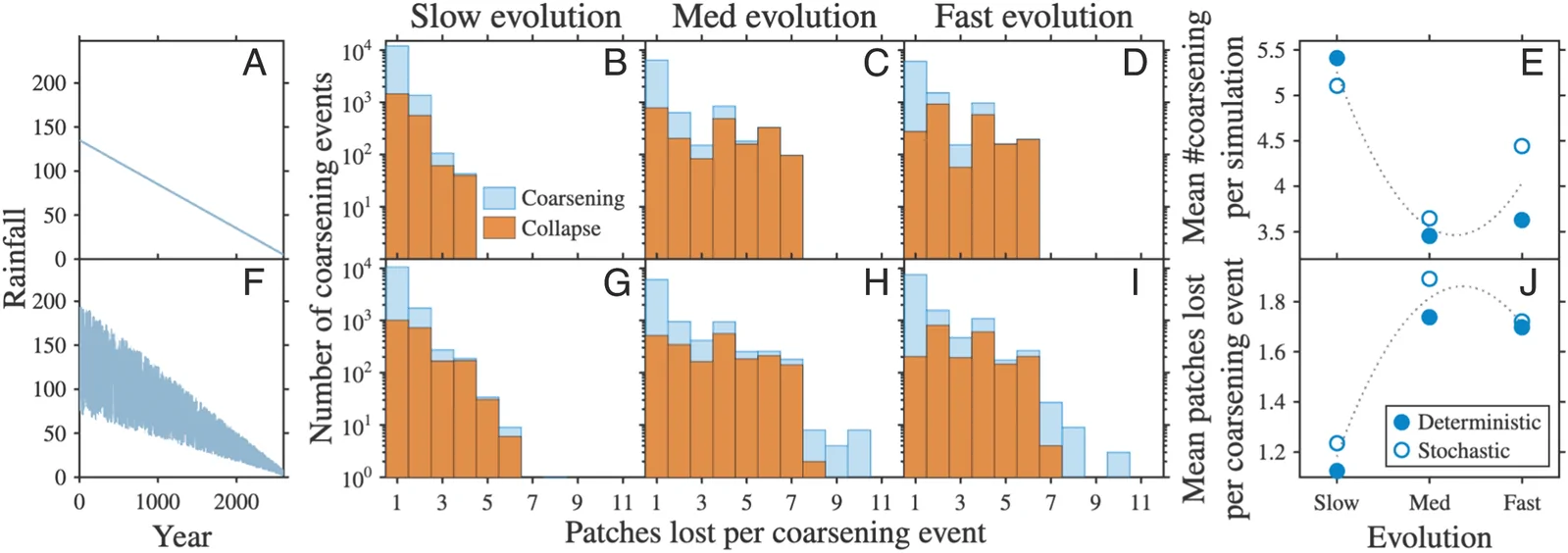
Rates of evolution affect frequency and magnitude of vegetation pattern transitions during drying. (*A*) Representative rainfall timeseries without stochasticity and (*B–D*) distribution of patches lost per coarsening event across 2,500 simulations under deterministic rainfall for increasing rates of evolution ($C_{\chi }=0.001,0.01,0.05$). A coarsening event is classified as a collapse when all patches disappear and total biomass remains zero. The model domain supports up to ~12 vegetation patches (*SI Appendix*, Fig. S1). As rate of evolution increases, (*E*) mean number of coarsening events per simulation decreases and then slightly increases. (*F*) Representative rainfall timeseries with stochasticity (coefficient of variation of 0.27) and (*G*–*I*) distribution of patches lost per coarsening event under stochastic rainfall for increasing rates of evolution. (*J*) Mean number of patches lost per coarsening event increases and then slightly decreases as rate of evolution increases. Parameter: $C_{P}$ = 0.05 mm y^−2^.

With the inhibitory effects of pattern formation on evolution weakened under stochastic rainfall, evolution feeds back on self-organization, altering both pattern plasticity and vegetation transitions. As plants evolve greater drought tolerance, patch shrinkage slows and the thinning phase is prolonged (Fig. 5). Thinner patches expose a greater fraction of edge plants to ambient drying, reinforcing selection for drought tolerance. The prolonged thinning can delay—or even suppress—patch coarsening (Fig. 5 and *SI Appendix*, Fig. S4), thereby reducing spatial plasticity: coarsening events become less frequent but more severe, with larger fractions of patches lost per event (compare Fig. 6 *B* and *C*).

Evolution also shapes spatial plasticity of vegetation patterns by altering the regularity of patch spacing (*SI Appendix*, Fig. S5). Spacing variance controls pattern transitions (28, 29): irregular spacing tends to lose patches one at a time—the one with the lowest biomass (least access to water)—producing incremental adjustments and gradual vegetation change (Movie S1). In contrast, regular spacing equalizes water access among patches but promotes synchronized, abrupt transitions, in which half the patches are lost simultaneously (Movie S1). By introducing trait heterogeneity among patches, evolution—especially when slow—increases spacing irregularity (*SI Appendix*, Fig. S6). Under slow evolution ($C_{\chi }=0.001$; ~1% of ecological rates), spacing variance (21.5 to 23.5, depending on rainfall stochasticity) more than doubles relative to both the no-evolution case (3.0 to 13.7, depending on fixed χ) and the no-local-adaptation case [~10.8, spatially uniform χ, as in Bera et al. (20)]. This elevated irregularity leads to frequent, small transitions (compare *SI Appendix*, Figs. S7 *C* and *D* and S8), a result robust to standardization by patch size.

As evolutionary rates increase, delays in coarsening strengthen (Fig. 5), whereas spacing regularity responds nonmonotonically, producing a nonlinear effect on spatial plasticity. Under slow evolution, spacing is highly irregular (variance 21.5 to 23.5), leading to frequent, incremental transitions (Fig. 6*B*). As evolution becomes more rapid ($C_{\chi }=0.01$; ~10% of ecological rates), trait convergence across patches regularizes spacing (8.1 to 10.8), shifting the system toward fewer but larger transitions (Fig. 6*C*). At still higher rates ($C_{\chi }=0.05$), regularity declines slightly (11.6 to 12.8), and transitions also become slightly more frequent and smaller (Fig. 6*D*). This reemergent irregularity may reflect the evolution of high drought tolerance (*SI Appendix*, Fig. S9*C*), which slows growth and mortality (Eq. **4**) and delays patch reorganization after coarsening (*SI Appendix*, Fig. S10). Consistent with this mechanism, fixed-trait simulations show higher spacing variance at χ=0.99 than at χ=0.01. Together, these results reveal a nonlinear relationship between evolutionary rate and pattern plasticity: slow evolution yields frequent, small adjustments, whereas more rapid evolution shifts dynamics toward fewer, larger transitions, with a slight reversal at high rates. Overall, evolution enhances plasticity (compare $\bar{f_{\mathrm{CRS}}}$ across scenarios with and without evolution in *SI Appendix*, Fig. S7). Under rapid evolution (~10% of ecological rates), however, up to ~40% of vegetation transitions involve loss of at least half the ecosystem biomass (Fig. 6 and *SI Appendix*, Fig. S7).

### Evolution Shapes Resilience Primarily via Spatial Plasticity.

**Fig. 7.**
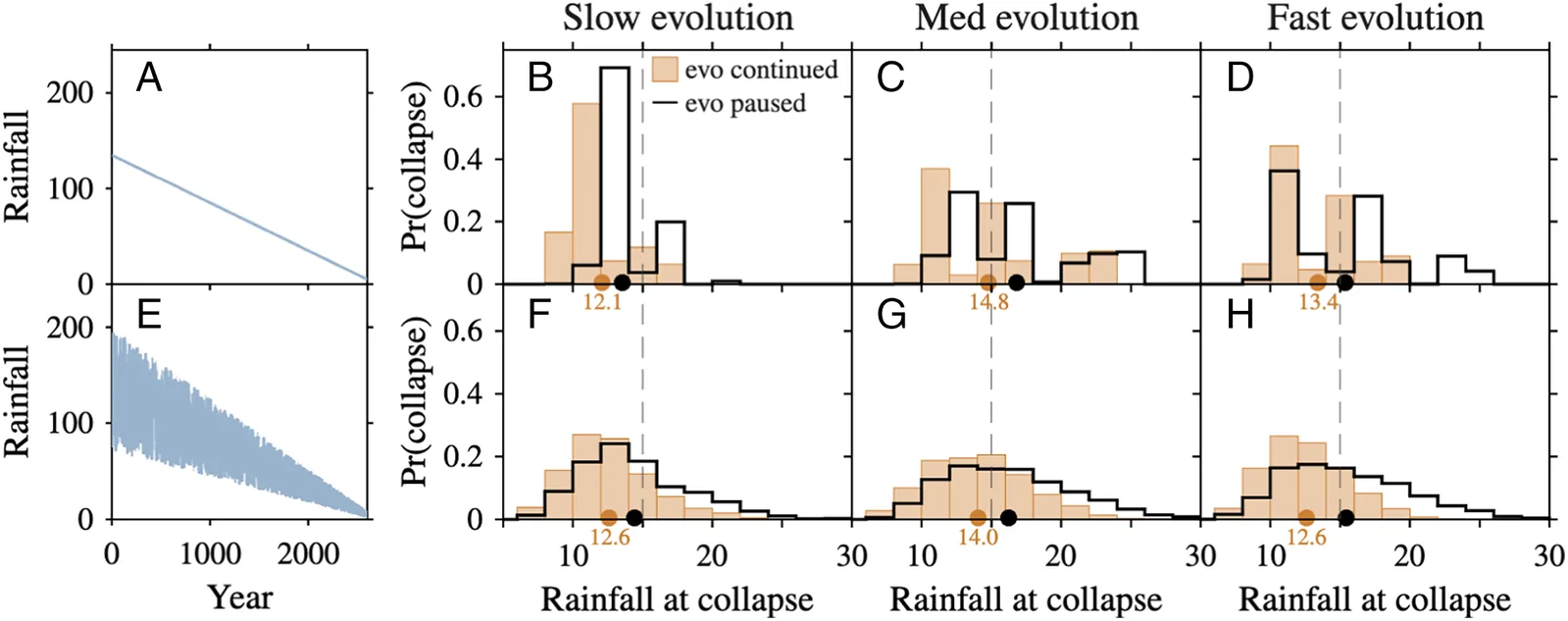
Rates of evolution affect ecosystem resilience under climatic drying. Resilience is measured as the rainfall threshold at which biomass declines to and remains at zero. (*A*) Representative rainfall timeseries without stochasticity. Distributions of collapse rainfall across 2,500 simulations under deterministic (*B*–*D*) rainfall. (*E*) Representative rainfall timeseries with stochasticity (coefficient of variation of 0.27) and (*F*–*H*) distributions of collapse rainfall under stochastic rainfall. Effects of evolution are assessed by comparing continuing-evolution (orange) and paused evolution (black)—in both cases, systems first reach equilibrium under constant rainfall at a given evolutionary rate (columns 2 to 4: $C_{\chi }=0.001,0.01,0.05$), after which drying begins with evolution either continuing or paused. Points denote mean collapse rainfall (numerical values labeled for the case of continuing evolution). Differences between scenarios are significant in all cases (p≈0, Mann–Whitney *U*, bootstrap, and permutation tests). Vertical dashed lines at *x* = 15 aid visual comparisons across panels. Collapse rainfall is calculated as a 3-y running mean at the time of vegetation collapse to reduce stochastic noise (results are robust to 2-, 5-, 10-y averaging). Parameter: $C_{P}$ = 0.05 mm y^−2^.

Evolution shapes dryland resilience through two pathways (*SI Appendix*, Fig. S11): indirectly, by altering the spatial plasticity of vegetation patterns, and directly, through adaptation. When both pathways are considered, evolution enhances resilience, quantified as the rainfall threshold for ecosystem collapse (Fig. 7; compare scenarios in which evolution is paused at the onset of drying versus allowed to continue; p<0.001, Mann–Whitney U, bootstrap, and permutation tests). Notably, evolving populations exceed the resilience of nonevolving populations fixed at maximal drought tolerance (χ=0.99) (*SI Appendix*, Fig. S12; p<0.001), indicating that increased drought tolerance alone cannot explain the effect. Instead, resilience is highest when vegetation patterns adjust flexibly through frequent, incremental pattern transitions under drying. This high spatial plasticity arises when evolution is slow ($C_{\chi }=0.001$; Fig. 7 and *SI Appendix*, Fig. S12*C*), even though drought tolerance is lower (*SI Appendix*, Fig. S9*A*), indicating that plasticity plays the dominant role.

Although evolution consistently enhances resilience, its effect depends nonlinearly on its rate (Fig. 7 and *SI Appendix*, Figs. S5, and S12). Slow evolution substantially increases resilience (*SI Appendix*, Fig. S12), but this benefit weakens under rapid evolution (orange points shift to higher rainfall from $C_{\chi }=$ 0.001 to 0.01; Fig. 7), because faster evolution delays coarsening and promotes more regular patterns, both of which limit incremental pattern adjustments under drying (Fig. 6*E*), thereby reducing resilience. At still higher rates ($C_{\chi }=0.05$), resilience partially recovers (Fig. 7*D*). This rebound likely reflects the evolution of high drought tolerance (*SI Appendix*, Fig. S9*C*), which slows growth and mortality and delays reorganization into regular patterns after coarsening. The resulting persistence of irregularity preserves pattern plasticity (Fig. 6*E*), boosting resilience. Together, these results show that evolution shapes resilience primarily by modulating the plasticity of vegetation patterns, whereas its direct effect through increased drought tolerance plays a secondary role.

### Stochasticity Preserves Evolutionary Effects but Directly Modifies Resilience.

Rainfall variability does not qualitatively alter how evolution affects pattern transitions or ecosystem resilience. Evolution continues to enhance resilience, with its magnitude varying nonlinearly with the rate of evolution (Fig. 7 and *SI Appendix*, Fig. S12; p≈0 across tests). This pattern is explained by corresponding nonlinear shifts in the spatial plasticity of vegetation patterns (Fig. 6*E*).

Despite this, rainfall variability directly reshapes the two pathways of resilience—adaptation and spatial plasticity (*SI Appendix*, Fig. S11*B*). Although stochasticity weakens constraints imposed by pattern formation on evolution, it ultimately slows adaptation (*SI Appendix*, Fig. S9), likely because fluctuating conditions disrupt consistent selection and increase the chance of maladaptation (44, 45). Consistent with this, the frequency of maladaptation during drying—instances in which populations evolve lower (higher) drought tolerance despite declining (increasing) rainfall—increases from <10% without stochasticity to 40% under stochastic rainfall. As a result, populations still trend toward greater drought tolerance during drying, but more slowly (*SI Appendix*, Fig. S9). Simulations under constant mean rainfall confirm this effect: stronger variability leads to lower equilibrium χ (*SI Appendix*, Fig. S13).

Rainfall variability also directly reshapes the second pathway of resilience—spatial plasticity—by amplifying both the frequency and severity of coarsening (Fig. 6). Although these metrics typically trade off, they rise together here because intermittent wet years under stochastic rainfall can temporarily promote vegetation recovery (*SI Appendix*, Fig. S14). Under realistic levels of rainfall variability (Fig. 6*F*) and evolutionary rates (~10% ecological rates), coarsening is ~5% more frequent than without stochasticity (Fig. 6*E*) and more extreme, with ~54% of events involving loss of half the patches. Collapses to bare soil state are likewise more catastrophic, with ~17% occurring directly from dense vegetation states (≥6 of a maximum of ~12 patches in the model domain). Together, rainfall stochasticity lowers the mean rainfall threshold for collapse while increasing the likelihood of collapse when conditions are still relatively favorable, extending the right-hand tail of the distribution (Fig. 7 and *SI Appendix*, Fig. S12).

## Discussion

Spatial self-organization and evolution are fundamental responses of ecosystems and organisms to environmental change (5, 15, 20); however, their interaction remains poorly understood (19, 46). Bera et al. incorporated evolution into models of vegetation pattern formation and showed that when evolution is much slower than ecological dynamics (spatial self-organization), the spatial plasticity of vegetation patterns constrains evolutionary adaptation (20). Here, by varying evolutionary rate, we show that this constraint persists even when evolution is as rapid as ecological dynamics (*SI Appendix*, Fig. S2*C*). The constraint does not arise from mismatched timescales. Rather, it reflects the fact that plants respond to local water availability—conditions stabilized by pattern plasticity (Fig. 3)—rather than directly to rainfall. As long as pattern formation persists, elevated soil moisture within vegetation patches relaxes selection for drought tolerance, thereby constraining evolution regardless of its rate.

Evolutionary homeostasis in spatially self-organized landscapes, while theoretically plausible, hinges on remarkably stable undercanopy soil moisture as rainfall declines (Fig. 4*A*) (20). In reality, undercanopy moisture fluctuates strongly in response to the interannual variability typical of arid and semiarid regions (21, 42, 47–49). This occurs because large rainfall variability exceeds the buffering capacity of vegetation repatterning and associated soil moisture modulation (48). As such, soil moisture tracks rainfall, allowing evolutionary adaptation to proceed. This is consistent with widespread evidence of rapid adaptation in dryland plants under contemporary climate change (17, 50) and over geological timescales. For example, the accelerated diversification of *Acacia*—a primary pattern-forming genus in Australia (51)—coincides with global aridification following the Middle Miocene Climatic Optimum (52). Under stronger rainfall variability projected with future warming (53), the inhibitory effects of pattern formation on evolution are likely to weaken further.

Evolution plays a dual role in shaping the resilience of spatially self-organized ecosystems (*SI Appendix*, Fig. S11): it enhances adaptation at the level of individual organisms while also altering system-level dynamics, both affecting resilience. The latter pathway can dominate. Critically, adaptations that increase individual fitness do not necessarily confer system-level resilience (13, 19). In our study, evolutionary adaptation to drought directly enhances resilience by allowing individual plants to persist under drier conditions, while also propagating through spatial self-organization to modify the spatial plasticity of vegetation patterns. We find that faster evolution, despite enhancing individual adaptation, can in fact reduce system resilience (Fig. 7 *B*, *C, F*, and *G*). Conversely, emergent system-level patterns can feedback to shape the local conditions organisms experience (19, 54), thereby steering evolutionary trajectory—but not necessarily toward adaptation to ongoing environmental change (19). For example, patch coarsening under drying can drive populations toward lower drought tolerance. Thus, processes in spatially self-organized systems can yield unexpected—and sometimes opposing—outcomes across levels of organization (19, 55), a feature likely common beyond drylands.

Many spatially self-organized systems play globally significant role in the fluxes and storage of water, sediment, and carbon (e.g., peatlands and coastal marshes) (56). Because resilience in these systems emerges from cross-scale interactions, where individual-level traits shape system-level dynamics, it is essential to incorporate traits and their rapid evolution into predictions, a key component largely overlooked in current models (19) (but see ref. 13). For example, in tidal marshes, plant traits affect sediment accretion locally while also reshaping the self-organization of channel networks; strikingly, this indirect, pattern-mediated pathway dominates the system-level sedimentation and thus marsh resilience under sea level rise (57). Neglecting rapid evolution may therefore lead to substantial errors in predicting resilience (13, 27). Many environmental drivers affect both evolution and spatial self-organization. Rainfall stochasticity, for instance, weakens evolutionary adaptation (*SI Appendix*, Fig. S13) while enhancing the spatial plasticity of vegetation patterns (Fig. 6). Similarly, the rate of climate change affects both pattern transitions (28, 29) and plant adaptive responses (58). Together, these results highlight the need to integrate cross-scale processes when forecasting resilience of spatially extended systems (10).

Beyond resilience to collapse, the regime of vegetation transitions is equally important, as sustained plant productivity underpins ecosystem functioning and human well-being. Recent theory predicts that pattern formation allows vegetation to evade catastrophic shifts through gradual, localized adjustments among multiple stable states under drying (10, 28). Yet empirical evidence suggests otherwise: ~50% of drylands globally exhibit abrupt shifts (30). Our results help reconcile this discrepancy by showing that abrupt transitions can remain common: across model scenarios spanning plausible rainfall variability and relative evolutionary-to-ecological rates, ~54% of transitions involve abrupt loss of at least half of total biomass. Rapid evolution—which is pervasive under climate change (50, 59)—and strong rainfall variability, characteristic of arid and semiarid regions, amplify small, gradual adjustments into large, abrupt transitions (Fig. 6). While this effect of rapid evolution has not been reported previously, the role of environmental stochasticity aligns with theory and observations showing more frequent abrupt shifts under stronger variability (30, 60). Higher rates of climate change may further amplify these transitions (29). Thus, although pattern formation may offer a pathway to evade tipping (10), future dryland change is likely to remain dominated by large-scale, abrupt shifts.

Our study moves beyond classical resilience theory, which focuses on a limited set of alternative stable states (8), to the emerging multistability theory for spatially extended systems, where ecosystems transition among coexisting patterned states (10). A central challenge is predicting which state an ecosystem will transition to, as this determines whether change is incremental or drastic (28, 29). Empirical observations highlight such variability: vegetation patterns in the Sahel have changed markedly in recent decades (61–63), whereas those in the Horn of Africa have remained largely stable (64). Although multiple factors likely contribute (64, 65), this contrast may also reflect differences in pattern regularity, with more irregular systems enabling incremental adjustments that maintain stability (28). Direct empirical tests of this link remain lacking. By identifying evolution as a driver of regularity, our results point to trait variation [e.g., specific leaf area, above- and belowground biomass ratios (38)]—increasingly accessible via remote sensing (66), as a tractable pathway for empirical validation (*SI Appendix*, section S2). Our results further highlight timescales, largely implicit in classical resilience theory, as a key dimension of multistable systems, where transitions depend critically on rates (29), e.g., those of climate change (28, 29), environmental variability (29), and evolution relative to ecological dynamics. These rates vary widely across systems. For example, pattern-forming species span grasses to shrubs and trees (51), encompassing a broad fast–slow plant economics spectrum (37) and distinct ecological and evolutionary rates. These systems may also differ widely in the traits under selection and in the ratio of patch size to individual plant size, which determines the number of individuals in a patch and may affect within-patch trait heterogeneity, edge effects, and ecoevolutionary dynamics. As a result, similar climatic forcing may yield gradual adjustment in some systems but abrupt collapse in others. Anticipating and managing dryland change will therefore require moving beyond universal predictions toward a context-dependent framework.

## Materials and Methods

### Rates of Rainfall Decline, Ecological Processes, and Evolutionary Dynamics.

Long-term observations of rainfall changes in global drylands show an average decline of 0.1 to 1.4 mm y^−2^, with substantial interannual variability (67–70). Projections suggest that annual rainfall in some dryland regions could decrease by 20 to 30% by the end of the 21st century, although other areas may become wetter (42). In our study, we considered a rainfall decline rate of 0.05 mm y^−2^. As we investigate ecosystems located in regions with the present-day rainfall between 15- and 135-mm y^−1^, a 0.05 mm y^−2^ decline rate corresponds to approximately 3 to 27% by the end of the century. We chose a relatively low rate because our simulations extend well beyond the 21st century, allowing mean rainfall to decline as low as 5 mm y^−1^ before the simulation is terminated. Under *C_p_* = 0.05 mm y^−2^, the normalized rate of rainfall decline, $P^{-1}\left|\frac{\mathrm{dP}}{\mathrm{dt}}\right|$, is on the order of 10^−4^–10^−3^ y^−1^.

In our model, the normalized rate of ecological change, $B^{-1}\frac{\mathrm{dB}}{\mathrm{dt}}$, is on the order of 10^−2^–10^−1^ y^−1^, corresponding to characteristic timescale of decades. Direct simulations corroborate that the reorganization of vegetation into a new pattern takes ~30 to 60 y, consistent with the decadal-scale dynamics inferred from empirical observations (71).

Under $C_{\chi }=0.001$, the normalized rate of evolution, $\chi^{-1}\frac{d\chi }{\mathrm{dt}}$, is on the order of 10^−4^–10^−3^ y^−1^, that is, about two orders of magnitudes slower than ecological change (~1% of ecological rates). When $C_{\chi }$ is increased to 0.01, the evolutionary rate is approximately 10% of ecological rates. These ratios were corroborated by direct simulations during the period when evolution is *not* inhibited by spatial self-organization (reactive thinning phase; Fig. 3) and in landscapes without pattern formation across a range of rainfall decline rates (*C_p_*) and levels of rainfall stochasticity (described below). According to a recent meta-analysis of rates of change in population size and phenotypic traits for a wide range of species and taxonomic groups, rates of phenotypic changes can be up to ~2/3 but on average 1/4 the associated rates of change in population size (11). We examined a wide range of $C_{\chi }$ values (0.001 to 0.05, corresponding to ~1 to 50% of ecological rates) to thoroughly assess the role of evolution and its rate.

### Rainfall Stochasticity in Drylands.

Drylands are characterized by pronounced climatic fluctuations, especially in rainfall, with relatively normal years often interspersed by severe droughts (72). Observed interannual variability in rainfall typically exhibits coefficients of variation (*CV*) between 30% and 80% and can reach up to 120% in some arid and semiarid regions (67–70). Because these estimates are generally based on relatively short observational periods (30 to 100 y), the *CV* over longer timescales—especially under future climate change, which is expected to amplify rainfall variability (53)—may be even higher.

To represent this variability, we modeled annual rainfall as a product of a deterministic long-term drying trend and stochastic noise. The deterministic component declines linearly over time, starting from an initial rainfall (*P_0_*, spanning 15 to 135 mm y^−1^ in 5 mm increments) and decreasing at a rate $C_{P}$, until reaching a lower bound of *P_min_* = 5 mm y^−1^. The stochasticity component is modeled as multiplicative uniform noise with a prescribed instantaneous *CV*. To ensure that the mean rainfall remains equal the deterministic mean, the stochastic multiplier $\varepsilon_{t}$ is drawn from a uniform distribution:

[6] $$\varepsilon_{t}∼U\left(a,b\right),a=1-CV\times \sqrt{3},b=1+CV\times \sqrt{3},$$

which ensures E[*ε*_*t*_] = 1 and SD[*ε*_*t*_] = *CV*. The resulting rainfall series is

[7a] $$P_{\mathrm{sto}}\left(t\right)=\varepsilon_{t}P_{\det}\left(t\right),$$

[7b] $$P_{\det}\left(t\right)=\text{max}\left(P_{0}-C_{P}t,P_{\min}\right),$$

where *t* is time in years since the onset of climatic drying. We considered three levels of stochasticity—instantaneous *CV* = 8, 27, and 54%—representing weak, moderate, and strong variability, respectively. The highest level (54%) corresponds to an upper-end scenario for drylands, yielding an overall *CV* (combining the drying trend and stochastic fluctuations) of approximately 63 to 81%, depending on $P_{0}$, $P_{\min}$, and $C_{P}$.

To assess the robustness of our findings, we also implemented three additional representations of rainfall variability: i) additive noise, ii) a two-state hidden Markov model representing “normal” and “drought” regimes (73), and iii) a first-order autoregressive model, i.e., AR(1) model. The latter two approaches capture temporal autocorrelation in annual rainfall, reflecting features such as multiyear droughts and regime switching—commonly observed in arid and semiarid regions (72). Nevertheless, the results reported in this paper are qualitatively consistent across all stochasticity representations and whether rainfall variability is imposed at annual or daily timescales.

### Numerical Solutions and Model Variants.

The model represents dryland ecosystems on flat, homogeneous terrain with uniform rainfall in space, where bare or sparsely vegetated soil is covered by physical or biological soil crusts that reduce the infiltration of surface water. Infiltration rates remain relatively high in densely vegetated areas, as plant roots increase soil porosity and soil crusts rarely develop there.

In addition to the full model (Eqs. **1**–**5**), we analyzed three other variants to isolate the effects of specific processes. i) No evolution variant: Eq. **5** was replaced by fixing χ as constant in both space and time; ii) No-pattern-formation variant: spatial self-organization was removed by setting f=1. And iii) well-mixed-population variant: evolutionary dynamics followed the formulation of Bera et al. (20):

[8] $$\frac{d\chi }{\mathrm{dt}}=\frac{C_{\chi }}{\int B^{2}\left(x\right)dx}\int B^{2}(x)\frac{\partial r}{\partial \chi }dx,$$

where the evolutionary dynamics are driven by the spatially averaged local selection gradient, weighted by the biomass squared so that areas of higher vegetation density contribute more strongly (20). In this variant, χ evolves over time, without explicitly accounting for its variability in space.

We also investigated the effect of alternative representation of competition by replacing the logistic growth term, $\left(1-\frac{B}{K}\right)B$ in Eq. **1**, with $\left(1-{\left(\frac{B}{K}\right)}^{\theta }\right)B$, where θ<1 (74). This formulation represents a rapid decline in per-capita growth at low population densities that gradually levels off at higher densities, whereas logistic growth assumes a linear decline with increasing density (74). Repeating the full set of analyses with this alternative growth function yields qualitatively identical results. For all these models, the spatial solution of the model was solved numerically using a finite difference method with periodic boundary conditions.

## Supplementary Material

Appendix 01 (PDF)

Movie S1. Changes of vegetation spatial patterns during climatic drying under two rates of evolution. (A) Slow evolution (*C_χ_* = 0.001), showing high spacing irregularity and coarsening that typically involve the loss of one patch at a time; (B) Faster evolution (*C_χ_* = 0.010), showing more regular spacing and coarsening events that often remove 50% of patches at a time. Results shown without rainfall stochasticity. Parameters: *C_P_* = 0.05 mm yr^−2^, initial *P* = 120 mm yr^−1^.

## Data Availability

Codes data have been deposited in GitHub: https://github.com/xdong05/evolution (75).
