# Supplementary material for: Evolution shapes ecosystem resilience through spatial self-organization
Source: Proc Natl Acad Sci U S A. 2026 Oct 12;123(42):e2536473123. doi: 10.1073/pnas.2536473123 (PMC13684302; doi:10.1073/pnas.2536473123)
Supplement: Supplementary file 1 [file pnas.2536473123.sapp.pdf]

PNAS

Supporting Information for

Evolution Shapes Ecosystem Resilience Through Spatial Self-Organization

Xiaoli Dong[1], Alexander J. Richardson[2], and Rachata Muneepeerakul[3]

1. Department of Environmental Science and Policy, University of California, Davis, California, USA, 95616
2. Department of Mathematics, University of California, Davis, California, USA, 95616
3. Department of Agricultural and Biological Engineering, University of Florida, Gainesville, Florida, USA, 32611

Corresponding author: Xiaoli Dong (Email: xldong@ucdavis.edu)

This PDF file includes:

Supporting Text S1. Deriving the evolution equation for χ

Supporting Text S2. Potential empirical tests of the model predictions

Figures S1 to S14

Table S1

Legends for Movie S1

SI References

Other supporting materials for this manuscript include the following:

Movie S1

Supporting Information Text

Section S1. Deriving the evolution equation for χ

Let $\chi(x,t)$ be the biomass-weighted mean trait and $B(x,t)$ the biomass density. Biomass follows

$$\partial_t B \;=\; r(B,W,H,\chi)\,B \;+\; D_B\,\partial_x^2 B, \qquad \text{with } r = c\Gamma\left(1-\frac{B}{K}\right)W - M. \tag{1}$$

We start from the trait density (total trait value) $Z := \chi B$, which is passively carried by B:

$$\partial_t(\chi B) \;=\; r(B,W,H,\chi)\,\chi B \;+\; D_B\,\partial_x^2(\chi B). \tag{2}$$

By the product rule,

$$\partial_t(\chi B) \;=\; B\,\partial_t\chi \;+\; \chi\,\partial_t B. \tag{3}$$

Next we substitute the biomass equation. From (1), $\partial_t B = r\,B + D_B\,\partial_x^2 B$. Plug this into (3) and set equal to the right-hand side of (2):

$$B\,\partial_t\chi \;+\; \chi\big[r\,B + D_B\,\partial_x^2 B\big] = r\,\chi B \;+\; D_B\,\partial_x^2(\chi B). \tag{4}$$

The terms $r\,\chi B$ appear on both sides of (4), so

$$B\,\partial_t\chi \;+\; \chi\,D_B\,\partial_x^2 B \;=\; D_B\,\partial_x^2(\chi B). \tag{5}$$

Apply the product rule to first and second derivatives:

$$\partial_x(\chi B) = \chi\,\partial_x B + B\,\partial_x\chi, \tag{6}$$

$$\partial_x^2(\chi B) = \partial_x\big(\chi\,\partial_x B + B\,\partial_x\chi\big) \tag{7}$$

$$= (\partial_x\chi)(\partial_x B) + \chi\,\partial_x^2 B + (\partial_x B)(\partial_x\chi) + B\,\partial_x^2\chi \tag{8}$$

$$= \chi\,\partial_x^2 B \;+\; 2\,(\partial_x B)(\partial_x\chi) \;+\; B\,\partial_x^2\chi. \tag{9}$$

Substitute (9) into (5):

$$B\,\partial_t\chi \;+\; \chi\,D_B\,\partial_x^2 B = D_B\Big[\chi\,\partial_x^2 B + 2\,(\partial_x B)(\partial_x\chi) + B\,\partial_x^2\chi\Big]. \tag{10}$$

Cancel the $\chi\,\partial_x^2 B$ terms that occur on both sides:

$$B\,\partial_t\chi \;=\; D_B\Big[2\,(\partial_x B)(\partial_x\chi) + B\,\partial_x^2\chi\Big]. \tag{11}$$

Divide by $B > 0$:

$$\partial_t\chi = D_B\left[\frac{2}{B}\,(\partial_x B)(\partial_x\chi) + \partial_x^2\chi\right]. \tag{12}$$

Rewrite (12) using $\partial_x \ln B = (\partial_x B)/B$:

$$\partial_t\chi \;=\; D_B\left[\partial_x^2\chi + 2\,\partial_x(\ln B)\,\partial_x\chi\right]. \tag{13}$$

Next we add directional selection along local selection gradient by including the additive genetic variance C_χ that captures the rate of evolution, which gives

$$\partial_t\chi \;=\; D_B\left[\partial_x^2\chi + 2\,\partial_x(\ln B)\,\partial_x\chi\right] \;+\; C_\chi\,\frac{\partial r}{\partial\chi}(B,W,H,\chi). \tag{14}$$

Note that $2D_B\,\partial_x(\ln B)\,\partial_x\chi$ acts as effective advection of the down / up biomass gradients of the trait.

Section S2. Potential Empirical Tests of the Model Predictions

Our model generates a set of mechanistic, testable predictions linking evolution, spatial self-organization, and ecosystem resilience. Specifically, the model predicts that evolution affects dryland resilience by altering trait heterogeneity among individual plants, which in turn modifies vegetation pattern irregularity and spatial plasticity. Although the complete causal chain remains challenging to test directly, several components are becoming empirically accessible.

A first and immediately testable prediction concerns the relationship between trait heterogeneity and regularity of vegetation patterns. Traits associated with adaptation to climatic drying (e.g., specific leaf area, leaf nitrogen content, and biomass allocation (1)) can increasingly be quantified using remote sensing (2). Likewise, regularity of vegetation patterns can be quantified from aerial and satellite imagery (3). Because both trait heterogeneity and vegetation patterns can be characterized at landscape scales across systems, the first link in the causal chain is becoming empirically testable: Do ecosystems with greater trait heterogeneity exhibit more irregular vegetation patterns, as predicted by the model? To minimize confounding effects, such comparisons should be conducted among systems in the same climatic region, thereby controlling for differences in climate change rates, which themselves affect pattern regularity (4), and among systems dominated by similar species or species belonging to the same functional group.

Testing the subsequent links in the causal chain—from vegetation pattern regularity to spatial plasticity, pattern transitions, and ecosystem resilience—requires long-term observations. The model predicts that ecosystems with more irregular patterns should undergo more frequent, smaller pattern adjustments during climatic drying, whereas ecosystems with more regular patterns should experience less frequent but more abrupt pattern transitions and ultimately collapse at higher rainfall levels. These predictions could be evaluated by monitoring vegetation spatial dynamics over decadal timescales across otherwise comparable patterned ecosystems.

Testing the model's central prediction—that evolution enhances resilience primarily through its indirect effect on spatial pattern plasticity rather than through increased drought tolerance of individual plants—is considerably more challenging. It requires comparing otherwise similar systems that differ in the rates at which drought-related traits evolve. Although evolutionary rates generally cannot be measured directly in natural drylands, independent evidence from common-garden experiments, reciprocal-transplant experiments, and other population genomic analyses could identify systems expected to differ in their rates of evolutionary responses (5, 6).

Within this framework, the model predicts that faster evolutionary responses (e.g., >10% of ecological rates) allow individuals to converge rapidly toward similarly high drought tolerance, thereby reducing among-individual trait heterogeneity and producing relatively regular patterns. In contrast, slower evolutionary responses (e.g., ~1% of ecological rates) maintain trait heterogeneity and generate more irregular patterns. With long-term observations, the model further predicts that faster-evolving systems will exhibit greater drought tolerance at the individual plant level but lower spatial pattern plasticity, resulting in less frequent but more abrupt vegetation transitions and collapse at higher rainfall thresholds. Conversely, slower-

evolving systems are predicted to maintain greater spatial plasticity through persistent trait heterogeneity, thereby delaying collapse despite lower drought tolerance of individual plants.

One possible approach would be to compare nearby patterned ecosystems dominated by closely related species, provided independent evidence indicates differences in the rates of evolution of drought-related traits. More generally, empirical observations showing that systems dominated by more drought-tolerant plants nevertheless exhibit lower resilience (e.g., collapse at higher rainfall thresholds) because of more regular vegetation patterns and reduced spatial plasticity would provide support for the model's central prediction that evolution can affect ecosystem resilience primarily through an indirect, pattern-mediated pathway rather than through enhanced drought tolerance of individual plants. Although such observations would not constitute definitive validation of the mechanism, they would provide an important empirical test of one of its most distinctive predictions.

Finally, observed trait variation may arise from phenotypic plasticity rather than genetic differentiation. Consequently, establishing the evolutionary basis of the proposed eco-evolutionary feedbacks will require complementary approaches such as common-garden experiments, reciprocal-transplant experiments, quantitative genetic analyses, or genomic methods (5, 6). These approaches would allow the contribution of genetically based trait variation to be distinguished from environmentally induced phenotypic plasticity.

Together, these approaches provide a roadmap for progressively testing the mechanistic predictions of the model, from currently feasible observations of trait heterogeneity and vegetation patterns to longer-term tests of vegetation transitions and ecosystem resilience, and ultimately to evaluating the model's central prediction that evolution affects ecosystem resilience primarily through indirect, pattern-mediated pathways.

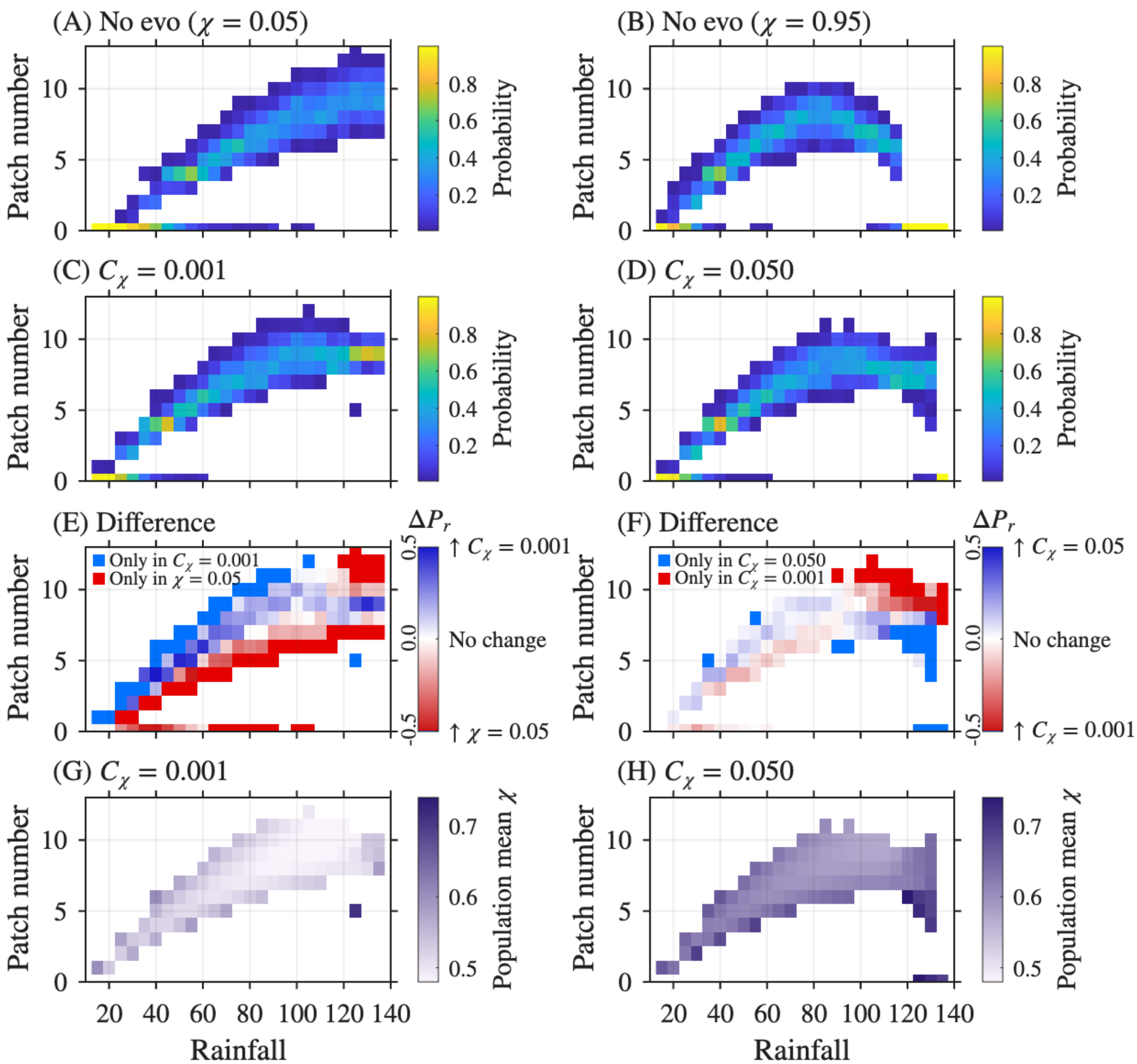

Figure S1. Multistability of vegetation spatial patterns and evolutionary states. (A-D) Probability of different stable spatial patterns, defined by the equilibrium number of periodic vegetation patches in a 300-cell model domain, across rainfall levels. Scenarios: no evolution with fixed $\chi = 0.050$ (A), no evolution with fixed $\chi = 0.95$ (B), and evolution with $C_\chi = 0.001$ (C) and $C_\chi = 0.050$ (D). For each rainfall level between 15 to 135 mm yr^{-1} (5 mm increment), 100 simulations were run from random initial conditions. Colors denote pattern probability, computed as normalized frequencies across 100 simulations. (E,F) Differences in pattern probability between scenarios, ΔPr: (E) $\Delta Pr = Pr(C_\chi = 0.001) - Pr(\chi = 0.05 \text{ fixed})$; (F) $\Delta Pr = Pr(C_\chi = 0.050) - Pr(C_\chi = 0.001)$. Bright blue and red indicate patterns unique to one scenario. (G,H) Multistability of the evolutionary stable population mean χ under two evolutionary rates.

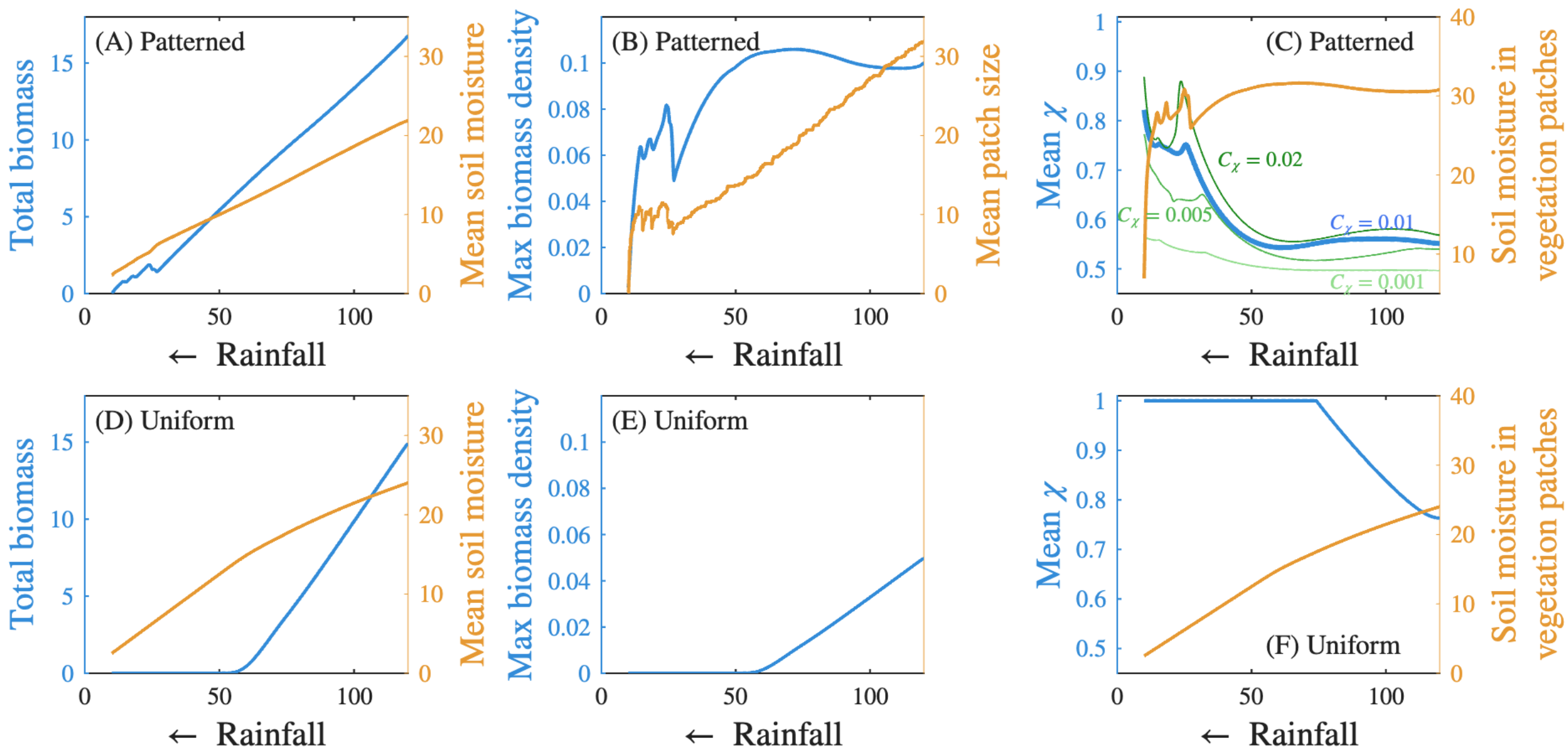

Figure S2. Responses of systems with and without pattern formation to climatic drying. (A-C) With pattern formation; (D-F) without pattern formation. (A,D) Total biomass and mean soil moisture decline linearly with drying in both cases. (B-C) With pattern formation, patch size continuously decreases as rainfall declines, while soil moisture and biomass density within vegetation patches and population mean χ remain approximately constant until rainfall drops to ~55 mm yr^{-1} (buffered thinning), after which they decline, triggering rapid adaptive evolution of χ (reactive thinning). Following coarsening, soil moisture and biomass density in vegetation patches and patch size increase, resetting the evolutionary state. Similar phase transitions occur across evolutionary rates. (E-F) Without pattern formation, soil moisture tracks rainfall, and χ increases monotonically (up to $\chi = 1$). Parameters: $C_\chi = 0.010$, $C_P = 0.1$ mm yr^{-2}, initial $P = 120$ mm yr^{-1}.

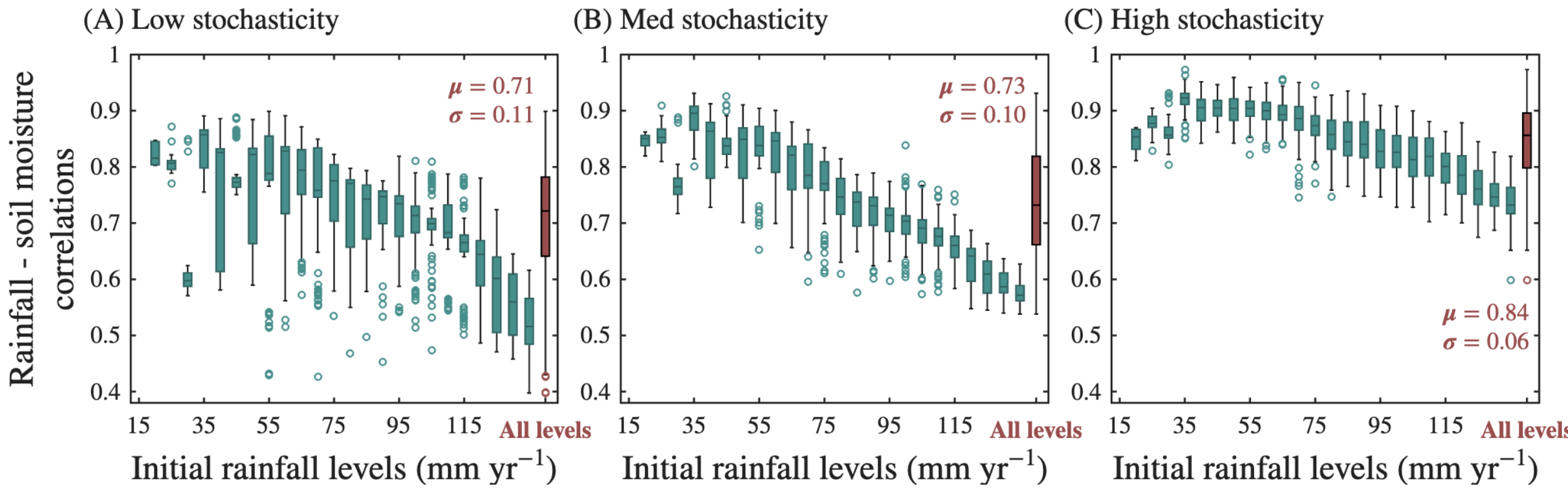

Figure S3. Rainfall stochasticity enhances correlation between rainfall and soil moisture within vegetation patches. Coefficient of variation (*CV*) of annual rainfall increases from 0.08 (A) to 0.27 (B) and 0.54 (C). Each panel shows correlations between rainfall and soil moisture during drying, based on simulations initialized at 25 rainfall levels with 100 simulations per level. The dark red boxes indicate correlations pooled across all rainfall levels (2,500 simulations), with mean (μ) and standard deviation (σ). Parameters: C_P = 0.05 mm yr^{-2}, $C_\chi = 0.010$.

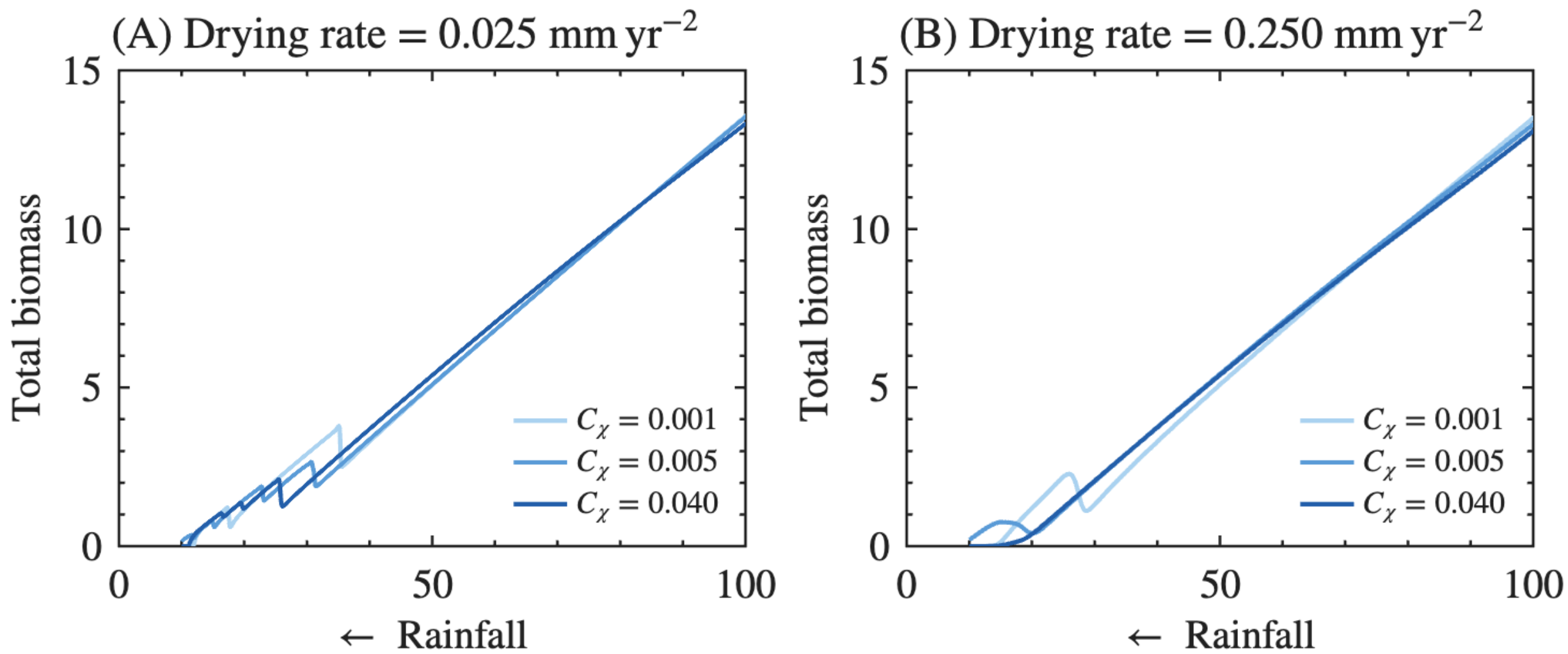

Figure S4. Evolution delays vegetation coarsening during drying. Coarsening occurs at lower rainfall levels when evolution is faster (higher C_χ). Under rapid climate change, coarsening can be fully suppressed (B). Results are shown without rainfall stochasticity. Parameter: initial P = 100 mm yr^{-1}.

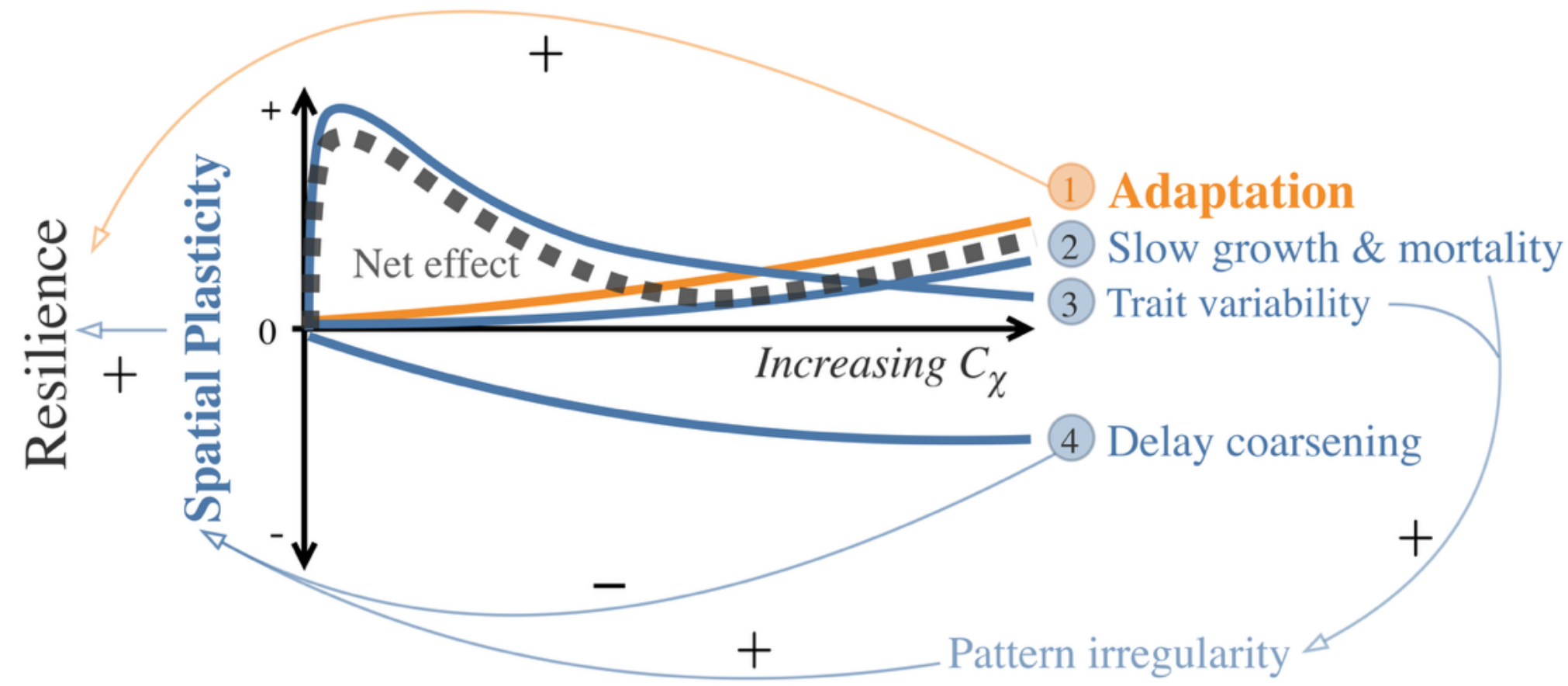

Figure S5. Pathways through which evolution affects resilience of patterned vegetation under drying. Evolution influences resilience both directly via adaptive change (orange; ①) and indirectly through its effect on the spatial plasticity of vegetation patterns (blue; ②, ③, ④). At slow evolutionary rates (small C_χ), increased trait variability leads to more irregular patch spacing, enhancing spatial plasticity and resilience; this effect weakens as C_χ increases (③). Faster evolution promotes rapid adaptation to drought, delaying coarsening of vegetation patches and reducing plasticity and resilience (④). High χ is associated with slow growth and mortality, slowing reorganization into regular patterns following coarsening (②) and thus increasing spatial plasticity, which can enhance resilience. The dashed gray curve indicates the net effect across pathways.

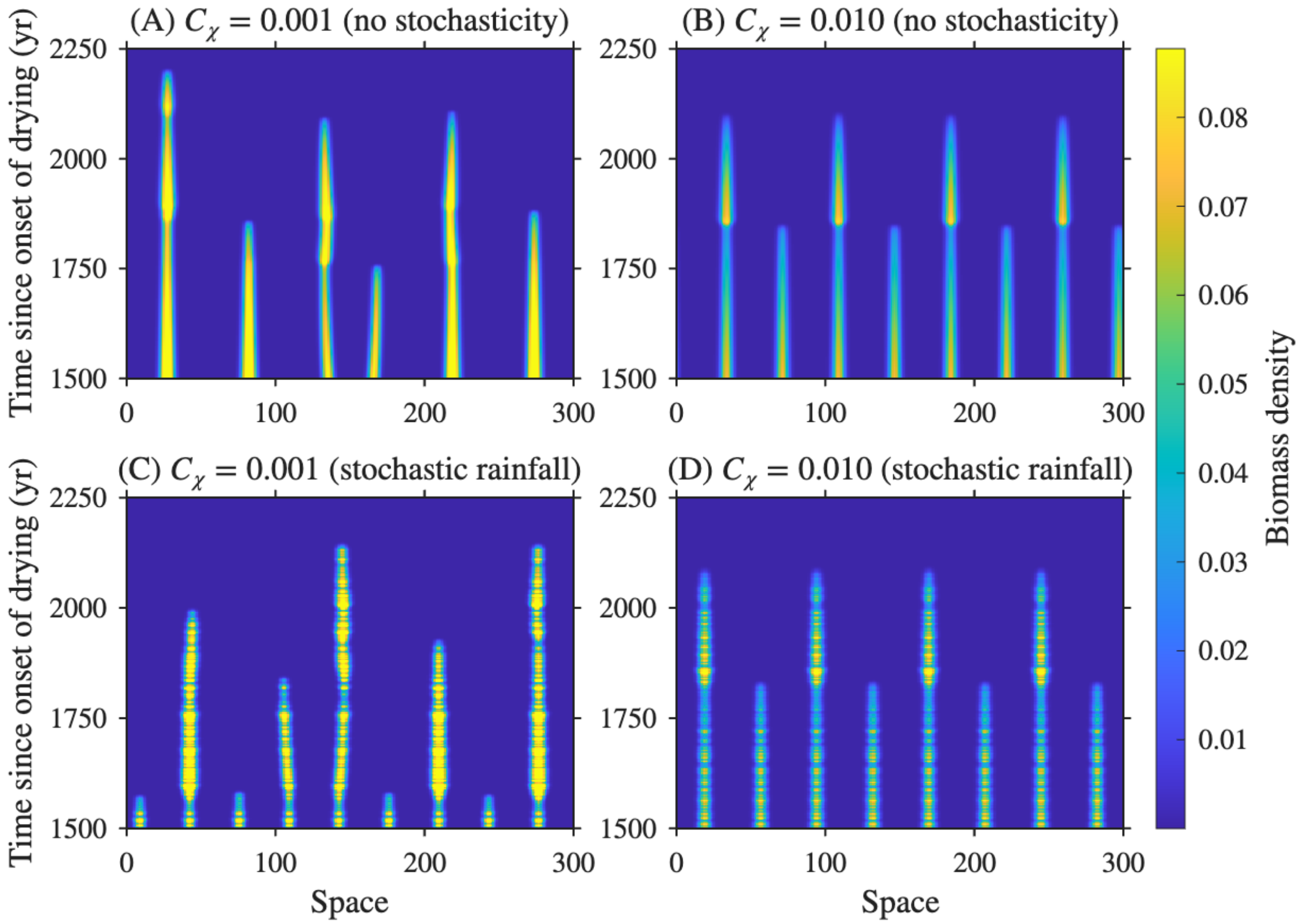

Figure S6. Slow evolution typically leads to sequential loss of vegetation patches, whereas rapid evolution typically leads to abrupt loss of 50% of patches per coarsening event. (A,B) Deterministic rainfall. (C,D) Stochastic rainfall (coefficient of variation of annual rainfall = 0.27). When rate of evolution is low (A,C), patch spacing is less regular and coarsening occurs via gradual loss of vegetation patches, typically one at a time, with relatively frequent events. In contrast, when evolution is rapid (B,D), spacing is more regular and coarsening events are less frequent but typically result in the loss of 50% of vegetation patches per event. Parameters: C_P = 0.05 mm yr^{-2}, initial P = 120 mm yr^{-1}.

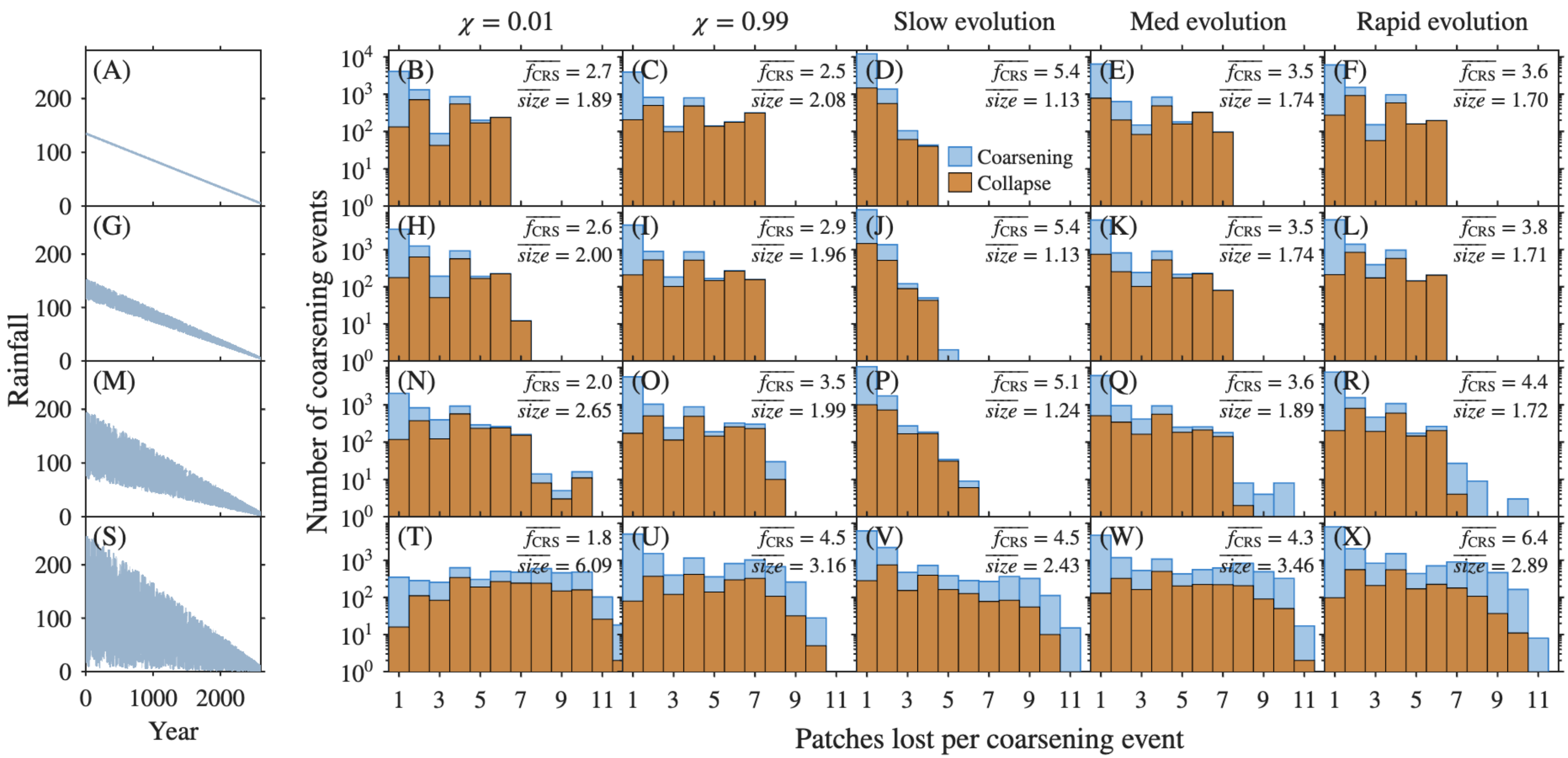

Figure S7. Effects of evolution and rainfall stochasticity on the frequency and magnitude of vegetation pattern transitions during climatic drying. The 300-cell model domain supports up to ~12 vegetation patches (Fig. S1). Columns 2-6 show the distribution of patch-number reductions per coarsening event across 2,500 simulations. A coarsening event is classified as a collapse when all patches disappear and total biomass remains zero. $\bar{f}_{CRS}$ denotes the mean number of coarsening events per simulation and $\overline{size}$ denotes the mean number of patches lost per event. Each simulation was run to equilibrium, then subjected to drying. We compare (1) cases without evolution at fixed drought tolerance ($\chi = 0.01$ *and* 0.99, columns 2-3) (2) different rates of evolution ($C_\chi = 0.001, 0.010, 0.050$; columns 4-6), and (3) deterministic (row 1) versus stochastic rainfall (coefficient of variation of annual rainfall = 0.08, 0.27, and 0.54; rows 2-4). Parameter: C_P = 0.05 mm yr^{-2}.

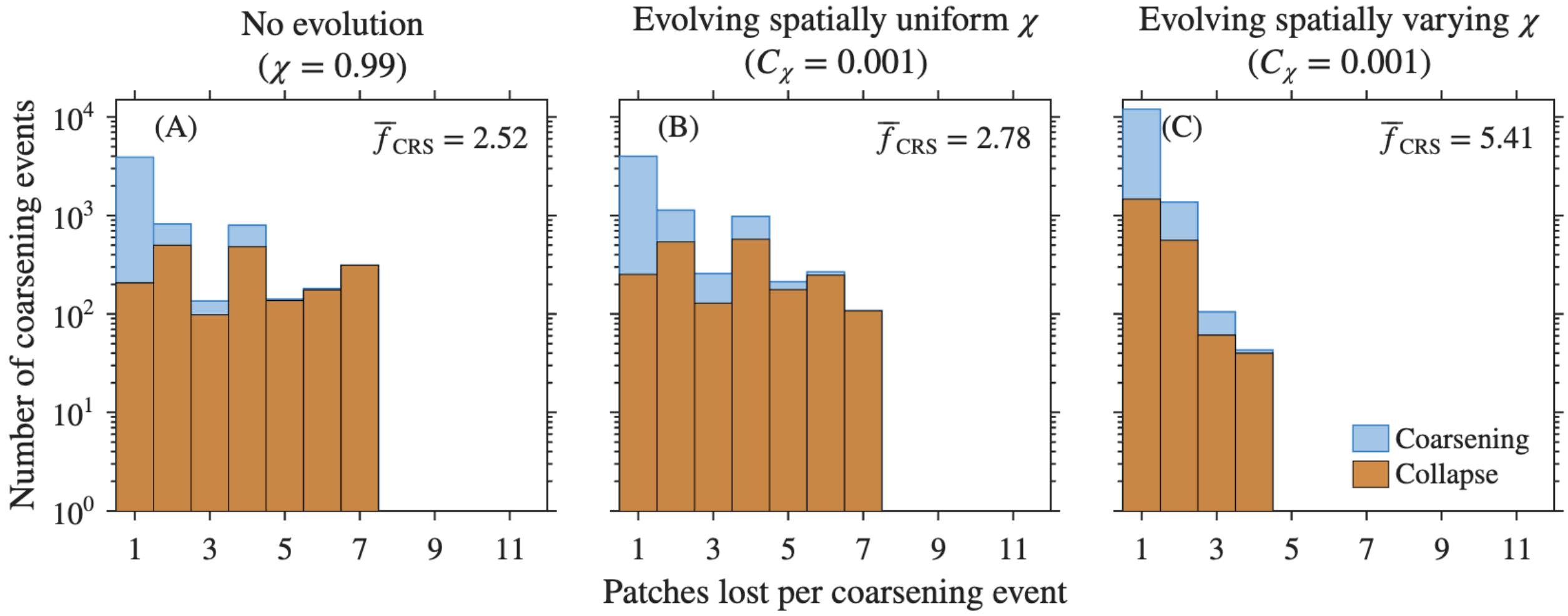

Figure S8. Local adaptation induces frequent small adjustments of vegetation patterns during climatic drying. (B) Results from the model without local adaptation, in which Eq. 5 is replaced by Eq. 8 and the trait χ is spatially uniform. (C) Results from the model with local adaptation, where χ varies spatially (Eq. 5). With local adaptation, slow evolution leads to more frequent (more than doubled) and smaller coarsening events (compare A and C). In contrast, without local adaptation, inducing slow evolution leaves the regimes of pattern transitions largely unchanged (compare A and B). $\bar{f}_{CRS}$ denotes the mean number of coarsening events per simulation, averaged across 2,500 simulations. Results are shown without rainfall stochasticity. Parameter: $C_P = 0.05$ mm yr^{-2}.

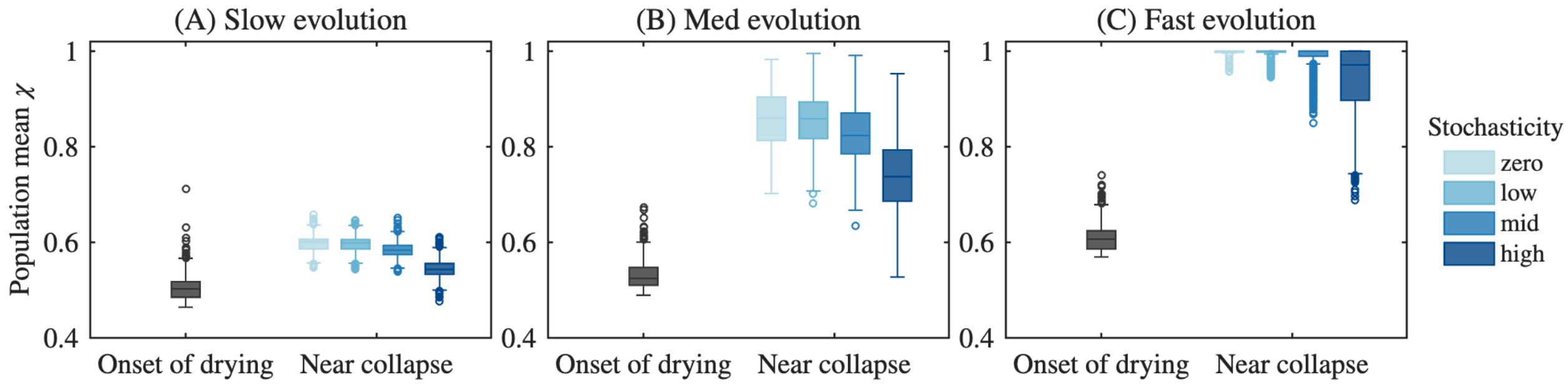

Figure S9. Rainfall stochasticity constrains evolution, whereas faster evolution enhances adaptive capacity. The model was first run to equilibrium under constant rainfall (15–135 mm yr^{-1}, in 5 mm increments; 100 simulations per level; 2,500 simulations total), yielding the population mean trait value (χ) at the onset of drying (dark gray boxes). Drying then proceeded. The evolutionary state near collapse (blue boxes) was quantified as the mean population χ over the five years proceeding vegetation collapse. We considered four drying scenarios—no, low (CV = 0.08), medium (CV = 0.27), and high (CV = 0.54) rainfall stochasticity—and three evolutionary rates: $C_\chi = 0.001$ (A), 0.01 (B), and 0.05 (C). Parameter: $C_P = 0.05$ mm yr^{-2}.

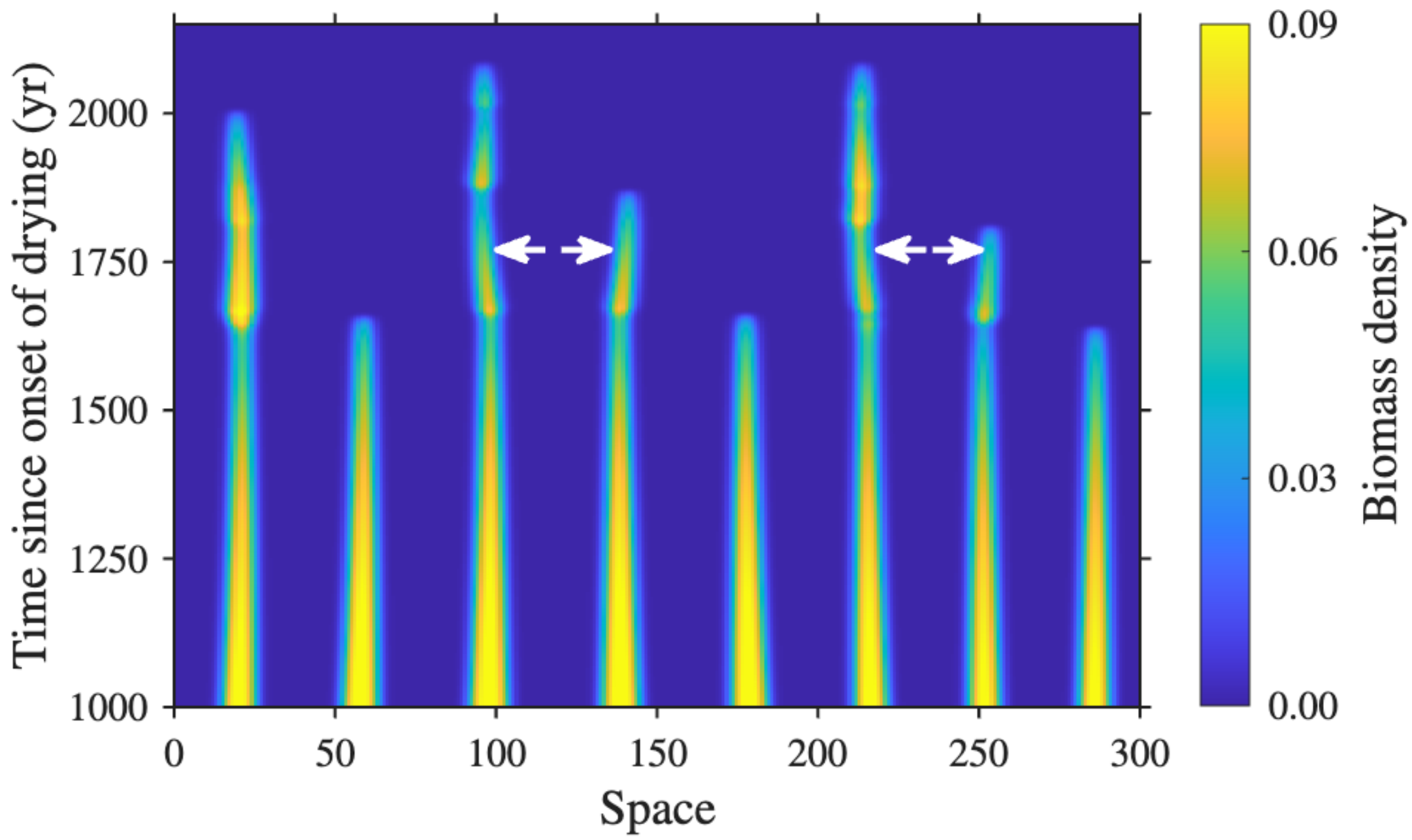

Figure S10. Adjustment of vegetation patch spacing into regularity following coarsening. Results are shown without rainfall stochasticity. White arrows indicate the direction of movement of vegetation patches. Parameters: C_P = 0.05 mm yr^{-2}, $C_\chi = 0.050$, initial P = 115 mm yr^{-1}.

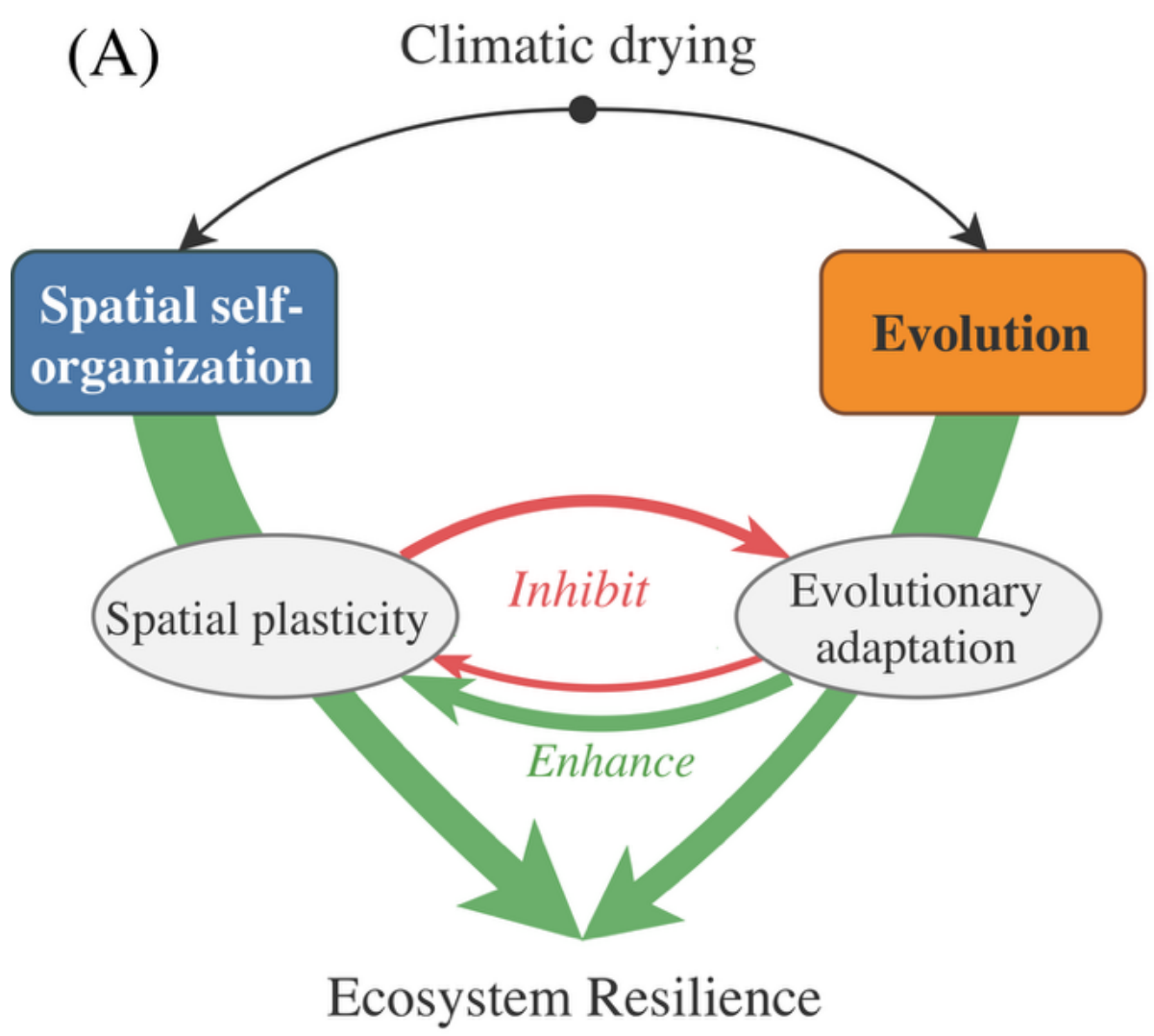

Figure S11. Schematic of how spatial self-organization and evolution shape ecosystem resilience under climatic drying, and the role of rainfall stochasticity. Red arrows depict inhibition and green arrows indicate enhancement; numbered circles denote differences between (A) and (B), i.e., effects altered by stochastic rainfall. Spatial self-organization and evolution affect resilience both directly and indirectly through their interaction. Rainfall stochasticity weakens the constraint of self-organization on evolution (①) but reduces adaptative evolution—and thus resilience—by directly impeding it (②③). Meanwhile, stochasticity directly increases the frequency of pattern transitions, thereby enhancing resilience (④⑤).

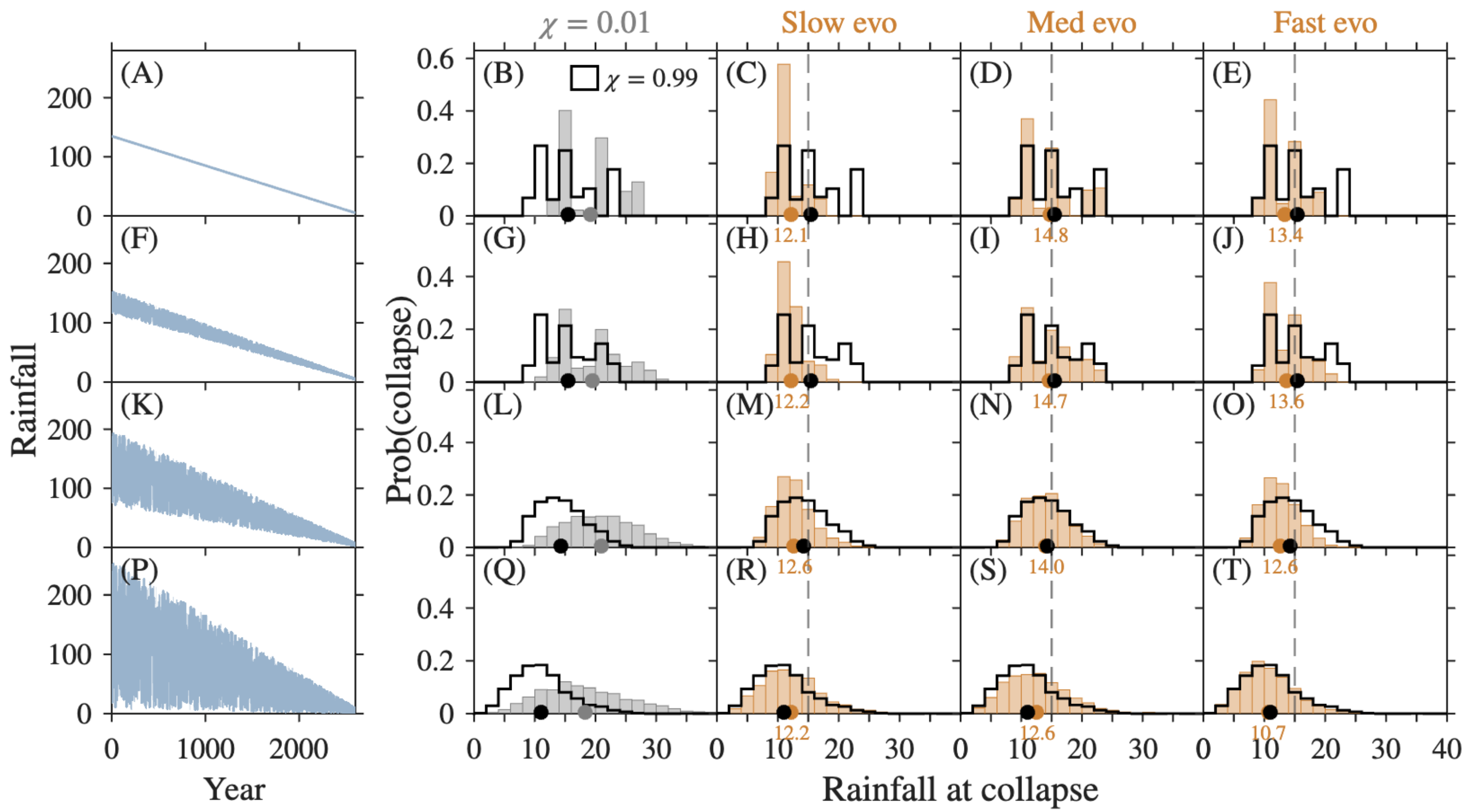

Figure S12. Resilience of evolving populations can exceed that of non-evolving populations fixed at maximal drought tolerance. Resilience is quantified as the threshold rainfall at which biomass declines to and remains at zero ("rainfall at collapse"). The first column shows representative rainfall time series illustrating different levels of stochasticity (rows 2-4: coefficient of variation of annual rainfall = 0.08, 0.27, and 0.54). Panels within each row show the corresponding resilience for that stochasticity level, represented by distributions of rainfall at collapse across 2,500 simulations. The second column compares two no-evolution cases with constant $\chi = 0.01$ (gray) and constant $\chi = 0.99$ (black). Columns 3-5 compare the constant $\chi = 0.99$ case with scenarios including evolution at different rates ($C_\chi = 0.001, 0.010, 0.050$; orange). Solid points along the x-axes indicate the mean rainfall at collapse across 2,500 simulations for each scenario, with colors corresponding to the associated distributions. Vertical dashed lines aid visual comparisons across panels. Parameter: $C_P = 0.05$ mm yr^{-2}.

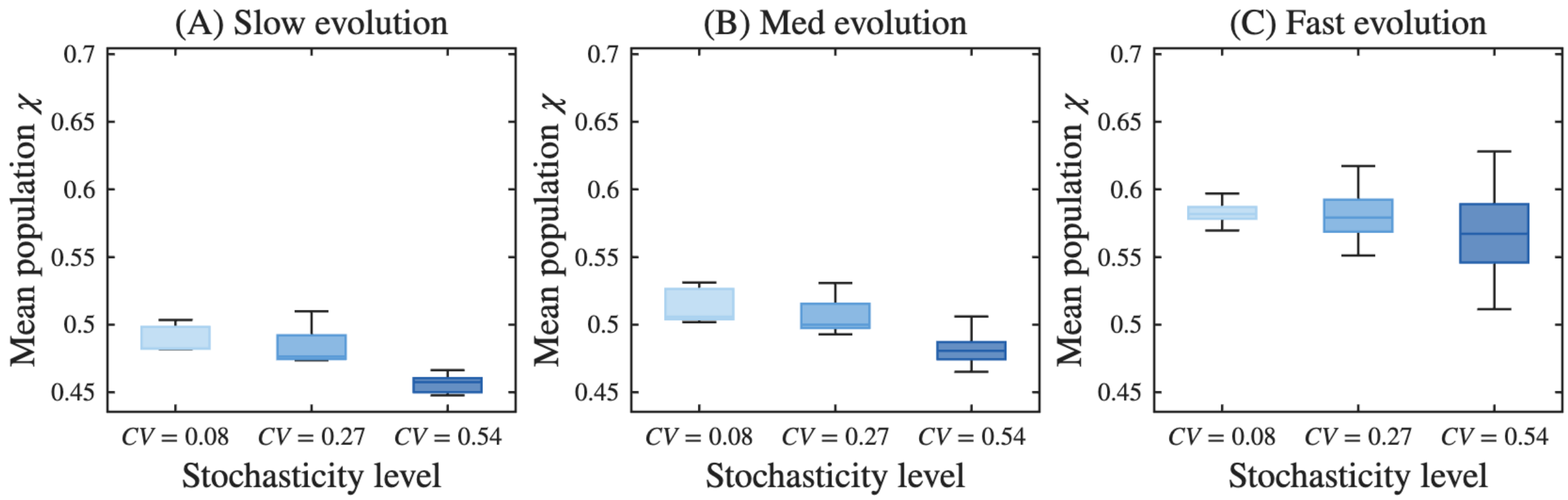

Figure S13. Rainfall stochasticity constrains evolution across rates of evolution. We fix mean rainfall at 90 mm yr^{-1} with no drying trend and vary levels of stochasticity, with the coefficient of variation (CV) of annual rainfall being 0.08, to 0.27, and to 0.54. (A-C) Results for different rates of evolution: $C_\chi = 0.001$ (A), 0.01 (B), and 0.05 (C). Boxplots show variations in the steady-state population mean χ across 100 simulations.

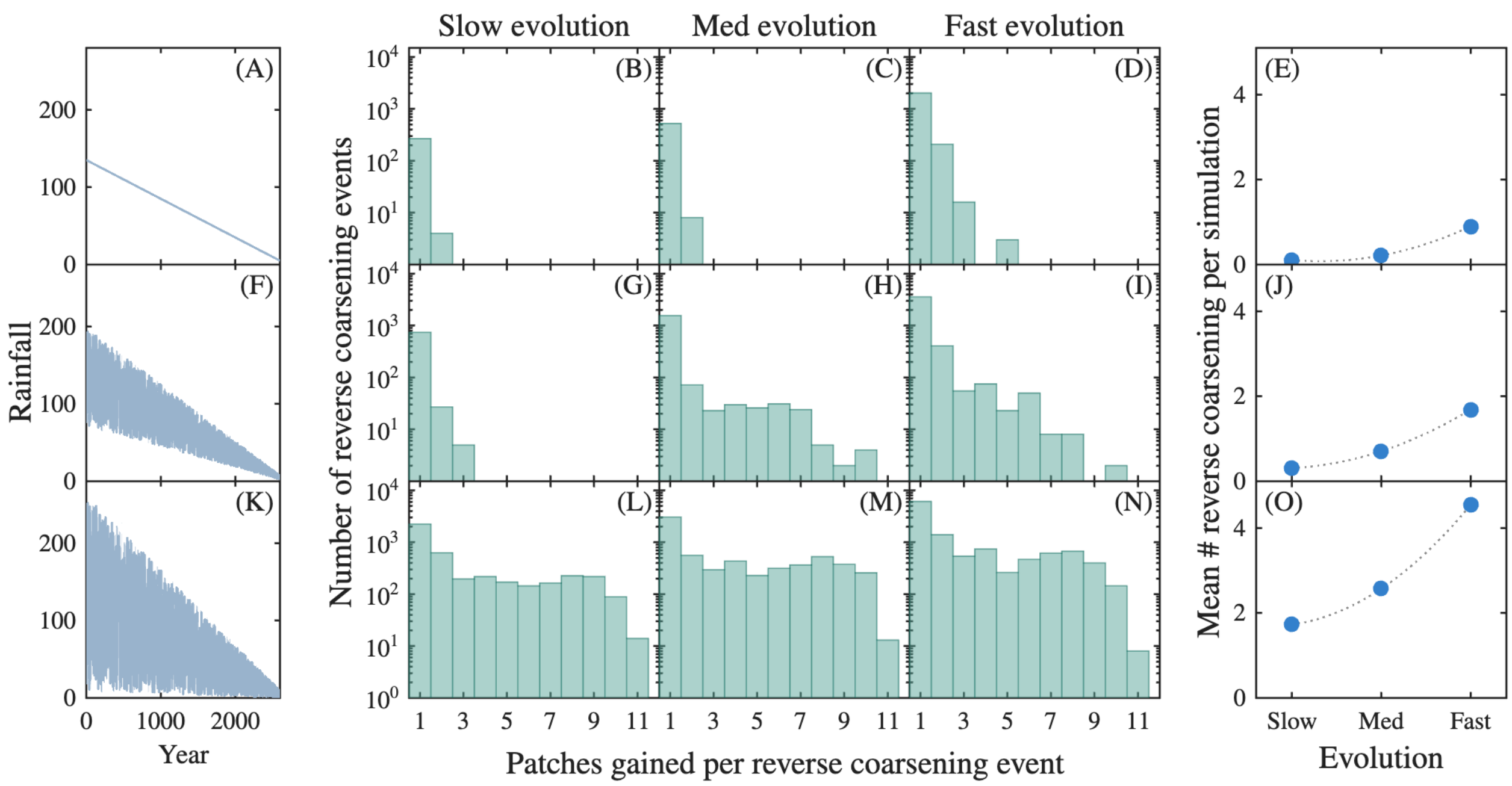

Figure S14. Frequency of reverse-coarsening increases with rainfall stochasticity and rates of evolution. Reverse-coarsening is defined as increases of vegetation patches in intermittent wet years within an overall drying trend. The first column shows representative rainfall time series illustrating different levels of stochasticity (rows 2-3: coefficient of variation of annual rainfall = 0.27, 0.54). Panels within each row show corresponding results: columns 2-4 display distributions of reverse coarsening event sizes; column 5 shows that the mean number of reverse-coarsening events per simulation across 2,500 simulations increases with evolutionary rates ($C_\chi = 0.001, 0.01, 0.05$). Parameter: C_P = 0.05 mm yr^{-2}.

Table S1. Model state variables, parameters, their descriptions, numerical values and units. Parameters values are after Bera et al. (2025) (7)

parameter	description	value	unit
B	biomass density	state variable	kg m^{-2}
W	soil moisture	state variable	mm
H	overland water level	state variable	mm
$\boldsymbol{\chi}$	trait describing growth-drought tolerance trade-off	state variable	dimensionless
c	water-to-biomass conversion efficiency (biomass gain per unit water uptake)	0.0016	kg m^{-2} mm^{-1}
$\boldsymbol{\Gamma}$	specific water uptake coefficient	20.0	m^2 kg^{-1} yr^{-1}
K	plant growth carrying capacity	variable with trait χ	kg m^{-2}
K_{min}	minimal carrying capacity	0.1	kg m^{-2}
K_{max}	maximal carrying capacity	0.6	kg m^{-2}
M	mortality rate	variable with trait χ	yr^{-1}
M_{min}	minimal mortality rate	0.5	yr^{-1}
M_{max}	maximal mortality rate	0.9	yr^{-1}
A	maximal infiltration rate	40.0	yr^{-1}
Y	weight constant that controls the influence of biomass on infiltration rate	0.9	dimensionless
f	infiltration-contrast parameter ($f \ll 1$ means high contrast between vegetated patches and bare soil)	0.01	dimensionless
Q	reference biomass at which infiltration is $\approx A/2$ with $f \ll 1$	0.06	kg m^{-2}
$\boldsymbol{L_0}$	evaporation rate in bare soil	4.0	yr^{-1}
$\boldsymbol{D_B}$	biomass dispersal coefficient	1.0	m^2 yr^{-1}
$\boldsymbol{D_W}$	soil moisture diffusion coefficient	10^2	m^2 yr^{-1}
$\boldsymbol{D_H}$	overland water diffusion coefficient	10^4	m^2 yr^{-1}
P	rainfall rate	variable	mm yr^{-1}
$\boldsymbol{C_\chi}$	additive genetic variance controlling rate of trait evolution	variable	dimensionless
$\boldsymbol{C_P}$	rate of rainfall decreases	variable	mm yr^{-2}

Movie S1. Changes of vegetation spatial patterns during climatic drying under two rates of evolution. (A) Slow evolution ($C_\chi = 0.001$), showing high spacing irregularity and coarsening that typically involve the loss of one patch at a time; (B) Faster evolution ($C_\chi = 0.010$), showing more regular spacing and coarsening events that often remove 50% of patches at a time. Results shown without rainfall stochasticity. Parameters: C_P= 0.05 mm yr^{-2}, initial P = 120 mm yr^{-1}.

SI References:

1. A. Eziz, *et al.*, Drought effect on plant biomass allocation: A meta-analysis. *Ecology and Evolution* **7**, 11002–11010 (2017).

2. L. Homolová, Z. Malenovský, J. G. P. W. Clevers, G. García-Santos, M. E. Schaepman, Review of optical-based remote sensing for plant trait mapping. *Ecological Complexity* **15**, 1–16 (2013).

3. R. Bastiaansen, *et al.*, Multistability of model and real dryland ecosystems through spatial self-organization. *Proceedings of the National Academy of Sciences of the United States of America* **115**, 11256–11261 (2018).

4. R. Bastiaansen, A. Doelman, M. B. Eppinga, M. Rietkerk, The effect of climate change on the resilience of ecosystems with adaptive spatial pattern formation. *Ecology Letters* **23**, 414–429 (2020).

5. V. L. Sork, Genomic Studies of Local Adaptation in Natural Plant Populations. *J Hered* **109**, 3–15 (2018).

6. P. De Villemereuil, O. E. Gaggiotti, M. Mouterde, I. Till-Bottraud, Common garden experiments in the genomic era: new perspectives and opportunities. *Heredity* **116**, 249–254 (2016).

7. B. K. Bera, O. Tzuk, J. J. R. Bennett, U. Dieckmann, E. Meron, Can spatial self-organization inhibit evolutionary adaptation? *J. R. Soc. Interface.* **22**, 20240454 (2025).
